# Adverse Effects of Vemurafenib on Skin Integrity: Hyperkeratosis and Skin Cancer Initiation Due to Altered MEK/ERK-Signaling and MMP Activity

**DOI:** 10.3389/fonc.2022.827985

**Published:** 2022-01-31

**Authors:** Marius Tham, Hans-Jürgen Stark, Anna Jauch, Catherine Harwood, Elizabeth Pavez Lorie, Petra Boukamp

**Affiliations:** ^1^ Department of Genetics of Skin Carcinogenesis, German Cancer Research Center (DKFZ), Heidelberg, Germany; ^2^ Department of Applied Tumor Biology, Institute of Pathology, University of Heidelberg, Heidelberg, Germany; ^3^ Institute of Human Genetics, University Heidelberg, Heidelberg, Germany; ^4^ Department of Dermatology, Royal London Hospital, Barts Health NHS Trust, London, United Kingdom; ^5^ Centre for Cell Biology and Cutaneous Research, Blizard Institute, Barts and the London School of Medicine and Dentistry, Queen Mary University of London, London, United Kingdom; ^6^ IUF–Leibniz Research Institute for Environmental Medicine, Düsseldorf, Germany

**Keywords:** skin cancer, organotypic skin cancer model, vemurafenib, cutaneous adverse effects, tumor invasion, matrix metalloproteinase, degradome, MEK inhibition

## Abstract

The BRAF inhibitor vemurafenib, approved for treating patients with BRAF V600E-mutant and unresectable or metastatic melanomas, rapidly induces cutaneous adverse events, including hyperkeratotic skin lesions and cutaneous squamous cell carcinomas (cSCC). To determine, how vemurafenib would provoke these adverse events, we utilized long-term *in vitro* skin equivalents (SEs) comprising epidermal keratinocytes and dermal fibroblasts in their physiological environment. We inserted keratinocytes with different genetic background [normal keratinocytes: NHEK, HaCaT (p53/mut), and HrasA5 (p53/mut+Hras/mut)] to analyze effects depending on the stage of carcinogenesis. We now show that vemurafenib activates MEK-ERK signaling in both, keratinocytes, and fibroblasts *in vitro* and in the *in vivo*-like SEs. As a consequence, vemurafenib does not provide a growth advantage but leads to a differentiation phenotype, causing accelerated differentiation and hyperkeratosis in the NHEK and normalized stratification and cornification in the transformed keratinocytes. Although all keratinocytes responded very similarly to vemurafenib in their expression profile, particularly with a significant induction of MMP1 and MMP3, only the HrasA5 cells revealed a vemurafenib-dependent pathophysiological shift to tumor progression, i.e., the initiation of invasive growth. This was shown by increased proteolytic activity allowing for penetration of the basement membrane and invasion into the disrupted underlying matrix. Blocking MMP activity, by the addition of ilomastat, prevented invasion with all corresponding degradative activities, thus substantiating that the RAS-RAF-MEK-ERK/MMP axis is the most important molecular basis for the rapid switch towards tumorigenic conversion of the HrasA5 keratinocytes upon vemurafenib treatment. Finally, cotreatment with vemurafenib and the MEK inhibitor cobimetinib prevented MEK-ERK hyperactivation and with that abolished both, the epidermal differentiation and the tumor invasion phenotype. This suggests that both cutaneous adverse events are under direct control of vemurafenib-dependent MEK-ERK hyperactivation and confirms the dependence on preexisting genetic alterations of the skin keratinocytes that determine the basis towards induction of tumorigenic progression.

## Introduction

Targeted therapy has revolutionized the field of medical oncology. Despite being highly successful in treating the specific malignancy, dermatologic toxicities (DT) are among adverse reactions of a variety of targeted therapies. Besides inflammatory dermatoses, i.e., papulopustular eruption, dermal hypersensitivity reaction (DHR), and photoreactivity, also hyperkeratosis and squamoproliferative lesions including actinic keratosis (AK), keratoacanthoma (KA), and cutaneous squamous cell carcinoma (cSCC) were described (for review, see 1 and references therein). Noteworthy, cutaneous epithelial proliferation, i.e., tumor formation, was particularly frequent with the BRAF inhibitors sorafenib and vemurafenib while substantially less frequent with EGFRi, MEKi, PI3Ki, or AKTi.

The oncogenic mutation V600E in BRAF protein accounts for 50%–60% of the somatic mutations in melanoma leading to constitutive activation of the protein kinase BRAF and downstream induction of the MAP kinase/ERK-signaling pathway with correlated melanomagenesis. Vemurafenib, a second-generation selective small molecule inhibitor of RAF, is highly potent in inhibiting the activation of this pathway in BRAF V600E-mutant melanoma cells and thereby is highly effective in combating the growth of metastases in the melanoma patients (for review, see 2). Unfortunately, acquired resistance and a high frequency of cutaneous adverse events, including the rapid development of cSCCs, impeded this treatment strategy. Accordingly, treatment was combined with a MEK inhibitor, with cobimetinib in case of clinical trials with vemurafenib. Concurrent administration of BRAFi and MEKi is now an established therapeutic protocol for the treatment of BRAF V600E-mutant metastatic melanoma and an adjuvant treatment in routine clinical practice. It is noteworthy, that cutaneous adverse events, including “keratinocytic proliferations” such as keratosis pilaris, i.e., small hyperkeratotic follicular papules (in up to 7% of patients), as well as actinic keratoses (AK), keratoacanthomas (KA), skin papillomas, and cSCCs emerged in 1%–2% of the patients (3). Thus, keratinocyte proliferation is still observed as a cutaneous adverse event even when treated with BRAFi+MEKi combination therapy, though at a much lower rate than in vemurafenib monotherapy which causes 6% hyperkeratosis, 8% KA, and 12% cSCC (4).

Generally, the development of cSCC is a multistep process with a latency period of several decades and therefore, cSCCs are frequent in elderly patients. cSCC are among the most common malignancies and characterized by a high load of UV-indicative mutations (5), as well as a high frequency of chromosomal aberrations with only few recurrent chromosomal aberrations (6, 7). Together, this makes these tumors genetically highly heterogeneous. The most frequent recurrent mutations and thus, implicated as driver genes in cSCCs, include NOTCH1/2, TP53, and CDKN2A while oncogenic ras, i.e., mutations in HRAS, Kras, or Nras are infrequent [3%–20% or less; (8) and references therein].

Sequencing of normal human eyelid skin (9) demonstrated a high frequency of mutations with a predominance of UV-indicative mutations (C>T mutations and high rates of CC>TT dinucleotide substitutions). We and others recently showed that normal sun-exposed skin contains numerous epidermal patches that stain positive for p53 protein and contain critically short, dysfunctional telomeres and which may be potential early precursors of skin cancer (7, 10). Cells of these patches contain mutations in multiple genes that are mutated also in cSCC with many of the mutations being subclonal in those lesions. Thus, various genetically altered cells exist in normal human skin, and it is tempting to propose that preexisting subpopulations contribute to the rapid development of skin cancer upon vemurafenib treatment.

Since vemurafenib is still an important component of targeted therapy for melanoma (11), there is a medical need for more extensive analysis of the pathogenesis of vemurafenib-dependent cutaneous adverse events. Considering their rapid appearance in the vemurafenib-treated melanoma patients, we expected to recapitulate the vemurafenib-specific phenotypes in long-term *in vivo*-like skin equivalents and to investigate the underlying regulatory switch responsible for the cutaneous adverse events. By utilizing keratinocytes representing different stages in the multistep process of skin carcinogenesis, we report that vemurafenib-associated MEK-ERK hyperactivation accelerates epidermal differentiation in different keratinocytes correlating with the vemurafenib-dependent adverse event of hyperkeratosis. In addition, we show a vemurafenib-dependent regulation of the degradome that is responsible for immediate initiation of invasive growth by the preneoplastic HrasA5 cells. This substantiates the hypothesis of a causal relationship of the tumorigenic shift observed *in vitro* and the rapid SCC development in Vemurafenib-treated melanoma patients.

## Results

### Vemurafenib Causes MEK-ERK Pathway Activation in Normal and Transformed Human Skin Keratinocytes

Vemurafenib abrogates RAF-MEK-ERK signaling in melanoma cells that harbor *BRAF V600E* mutations while causing pathway hyperactivation in wildtype melanoma cells (12, 13). MEK-ERK hyperactivation is also seen in epithelial *BRAF* wildtype cells, e.g., the human HaCaT keratinocytes and different human cSCC cells (14).

Thus, we asked how different human keratinocytes would respond to vemurafenib. For this, we investigated normal human epidermal keratinocytes (NHEK) as well as keratinocytes from our isogenic human skin cancer model (15); the HaCaT cells as well as their Hras-containing tumorigenic variants, the benign tumorigenic HrasA5 cells and the malignant tumorigenic HrasII4 cells. Using a phosphorylation-specific Western blot analysis, we investigated the time-dependent pathway activation upon treatment with 1 µM vemurafenib.

**Figure 1:**
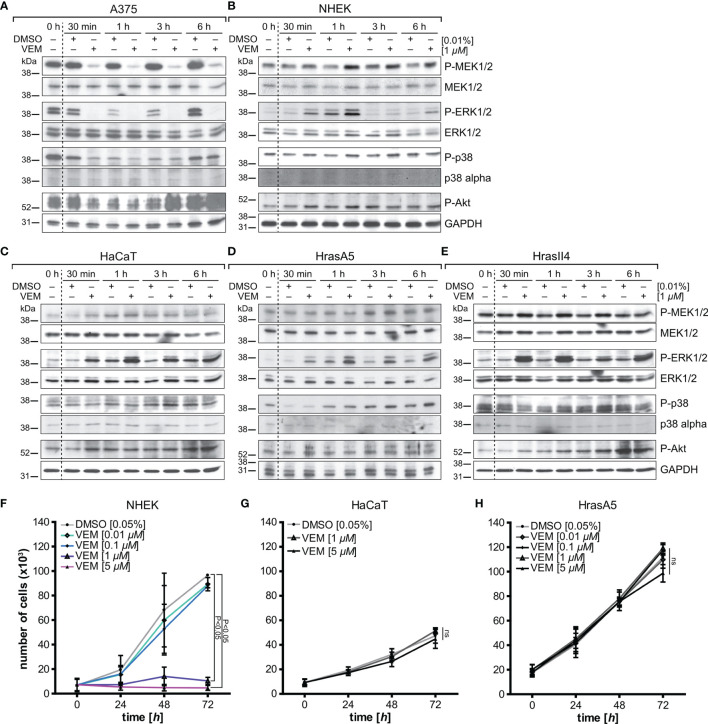
Vemurafenib-dependent pathway regulation and proliferation. A375 (BRAF mutant) melanoma cells **(A)** and BRAF wildtype keratinocytes, represented by normal human keratinocytes (NHEK) **(B)**, HaCaT **(C)**, the premalignant HrasA5 **(D)**, and the malignant HrasII4 cells **(E)**, were incubated with either DMSO (solvent control) or vemurafenib (1 µM) for a period of up to 6 h, and protein expression of MEK/P-MEK, ERK/P-ERK, p38/P-p38, and Akt/PAkt was examined. Opposing effects were seen for P-MEK and P-ERK when comparing the A375 melanoma cells (*BRAF-V600E* mut) and the human keratinocytes. P-p38 and P-Akt were inhibited transiently in the A375 cells, both in control and vemurafenib-treated cells, but did not seem to be regulated in the human keratinocytes. GAPDH was used as loading control in all immunoblots. To study the effects on proliferation, NHEK **(F)**, HaCaT **(G)**, and HrasA5 cells **(H)** were treated with different concentrations of vemurafenib (0.01–5 μM) for a period of up to 72 h, and proliferation was determined at 24, 48, and 72 h by measuring fluorescence intensity (SyBr green proliferation assay). Statistical significance was calculated by two-way ANOVA and Bonferroni posttest (*n* = 2, mean ± SD, two-way ANOVA + Bonferroni posttest; ns, not significant).

In *BRAF V600E-*positive A375 melanoma cells, which we included as control, vemurafenib rapidly (within 30 min) abolished the high basic levels of phosphorylated MEK1/2 and ERK1/2 (**Figure 1A**). In contrast, two different strains of the *BRAF* wildtype NHEKs showed similar results to the different HaCaT variants, HrasA5 and HrasII4 cells, as vemurafenib caused activation of the MEK-ERK pathway, indicated by an increase in P-MEK1/2 and even more so in P-ERK1/2 (**Figures 1B–E**). Interestingly, activation appeared transient in the NHEK, while being long lasting in the HaCaT cells (p53 mut) and the Hras oncogene-containing variants (p53mut/Hras+).

To confirm whether vemurafenib would activate also additional signaling cascades, we analyzed for coactivation of the MAPK-p38 as well as PI3K-PTEN-Akt pathway. In the melanoma cells, Pp38 and P-Akt were temporarily reduced (30 min to 3 h post treatment) (see **Figure 1A**). As reduction was seen in the untreated and vemurafenib-treated cultures, a vemurafenib-specific regulation was unlikely. In the keratinocytes, the level of Pp38 remained largely unaffected except for HrasA5 where P-p38 was temporarily lowered (first 30 min). Likewise, P-Akt showed no major regulation; if at all, there was a slight increased over control.

Together, this shows that vemurafenib causes rapid activation of the MEK-ERK pathway while having little effect on the p38 and Pi3K-PTEN-Akt pathways, thereby making MEK-ERK hyperactivation the major vemurafenib-dependent regulatory consequence in the human skin keratinocytes irrespective of their state of transformation.

### Vemurafenib Does Not Increase Proliferation in Cultivated Human Skin Keratinocytes

Commonly, MEK-ERK activation is linked to growth (16) and hyperactivation of the MEK-ERK cascade was supposed to be a major signaling pathway triggering proliferation (17). Increased proliferation was suggested also for HaCaT cells treated with a “low” vemurafenib concentration (2 µM) (18). To address how vemurafenib would affect growth of our different keratinocyte variants, we exposed NHEK, HaCaT, and HrasA5 cells to 0.1 up to 5 µM vemurafenib and determined their growth kinetics over the following 3 days (**Figures 1F–H**). Instead of increased proliferation, vemurafenib-treated NHEK showed the same growth rate as the nontreated control cells at low concentrations (0.1 and 0.01 µM). At higher concentrations (1 and 5 µM), vemurafenib induced significant growth inhibition. HaCaT and HaCaT-rasA5 (HrasA5) cells appeared less sensitive. Even the highest concentration of vemurafenib (5 µM) did not affect short-term growth in conventional cultures. Only upon long-term treatment (>1 week) with 5 µM of vemurafenib, a 50% growth reduction was seen for the HaCaT cells while the HrasA5 cells were not affected at all (data not shown).

Taken together, vemurafenib-induced MEK-ERK hyperactivation did not improve growth in any of the keratinocytes. Instead, we found acute growth inhibition for 1 and 5 µM vemurafenib for the NHEK and delayed growth restraints for the HaCaT keratinocytes. The premalignant HrasA5 cells remained unperturbed, indicating a transformation stage-specific loss of sensitivity for vemurafenib-induced growth restraints in the human keratinocytes.

### Vemurafenib Does Not Confer Chromosomal Instability But Rather Promotes a Genetic Drift

The rapid development of cSCC in vemurafenib-treated melanoma patients and the evidence that BRAF inactivation drives aneuploidy (19) may suggest vemurafenib-dependent genomic instability. Alternatively, vemurafenib may select for and promote preexisting subpopulations. To address the role of vemurafenib in genomic instability, we utilized the nontumorigenic HaCaT cells. Like numerous cells present in sun-exposed skin, they carry UV-type-specific p53 mutations, thus suffering from lack of the property of p53 to induce DNA repair and cell cycle arrest. Nevertheless, they are stably nontumorigenic and remain as a superficial epidermis-like epithelium upon long-term propagation as skin equivalents in 3D organotypic cultures or xenotransplants in mice (20, 21).

To determine whether and how vemurafenib would contribute to chromosomal instability, we performed multiplex fluorescence *in situ* hybridization (M-FISH) of HaCaT cells treated with 1 or 5 µM vemurafenib for 5 weeks. We show that neither dose resulted in gross chromosomal changes. Comparison of numerical aberrations for individual chromosomes demonstrated a very similar profile with only few changes (**Supplementary Figure S1A**). However, when comparing the aberration profile of control and vemurafenib-treated HaCaT cells for the distribution of subpopulations, vemurafenib provoked a shift in the dominance of preexisting subpopulations (**Supplementary Figure S1B**). In particular, we detected dominance for a dose-dependent gain of i(1q), carrying genes such as *S100* genes, *RASSF5*, *MAPKAPK2*, *TP53BP*, *WNT3A*, and *WNT9A*, and dose-dependent loss of i(17p), containing genes such as *TP53*, *MAP2K4*, *MAPK7*, or *RASD1*. We also found a selection for an unbalanced translocation chromosome der(2)t(2;8), leading to copy number gain of 8q harboring the *cMYC* gene—a cytogenetic aberration frequently associated with cSCCs (6, 22).

Together, this genetic analysis suggests that vemurafenib is not a potent inducer of genetic alterations. Instead, vemurafenib may provide a selective advantage for specific subpopulations and those with, e.g., excessive cMyc that are also able to respond with tumorigenic/invasive growth.

### Vemurafenib Alters Gene Expression of Keratinocytes

To determine whether vemurafenib would also affect the expression profile of the skin keratinocytes, we performed RNA expression analyses for NHEK, HaCaT, and HrasA5 cells by selecting a panel of genes, including epidermal differentiation markers, pathway-indicative, and invasion-related genes. For this, the different keratinocytes were treated with 1 and 5 µM vemurafenib and expression was analyzed by qRT-PCR after 8 and 24 h.

**Figure 2:**
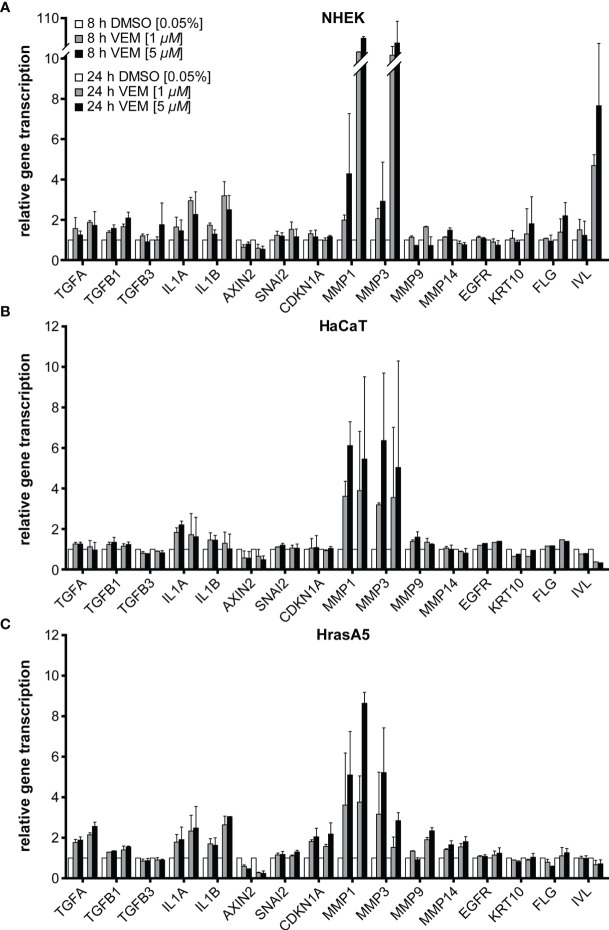
Vemurafenib preferentially targets the degradome. Vemurafenib-altered expression profile of NHEK **(A)**, HaCaT **(B)**, and HrasA5 cells **(C)** after 8 and 24 h of vemurafenib treatment. All human keratinocytes show upregulation of *IL1A* and *IL1B*, with HaCaT cells being the least regulated, as well as a strong induction of *MMP1* and *MMP3* while *MMP9* and *MMP14* remain largely unaffected. In addition, epidermal differentiation markers become upregulated in NHEK only. Normalization was performed using GAPDH as house-keeping gene and foldchanges were expressed by comparing 1 or 5 µM vemurafenib treatment of NHDF to DMSO stimulation, respectively. *n* = 2, mean ± SD.

Concerning epidermal differentiation, a minor induction of involucrin was seen in NHEK after 8 h, which increased considerably after 24 h. Likewise, keratin 10 (*KRT10*) and filaggrin (*FLG*) were upregulated after 24 h and the degree of regulation appeared dose-dependent (**Figure 2A**). None of those genes were regulated in HaCaT or HrasA5 cells during the first 24 h (**Figures 2B, C**). Only upon long-term treatment of the HaCaT cells with 5 µM vemurafenib for 4 and 8 weeks we saw an increase in the transcription of *KRT10* and *FLG* (data not shown). This suggests that induction of differentiation is rapid and direct in NHEK while delayed and potentially indirect in the transformed keratinocytes.

In addition, we found a 2- to 3-fold induction of the interleukins *IL-1α* and *IL-1β* (**Figure 2A**), factors known to act on the dermal fibroblasts in a paracrine stimulatory loop by inducing, e.g., keratinocyte growth factor (KGF alias *FGF7*) and granulocyte macrophage colony-stimulatory factor (GM-CSF alias *CSF2*). These in turn support epidermal growth and differentiation (23, 24). Transforming growth factor alpha (*TGF-α*) reached a 2-fold increase in NHEK and HrasA5 cells while transforming growth factors *TGF-β1* and *TGF-ß3* remained largely unaffected. Likewise, *AXIN2* (Wnt/ß-catenin pathway), *CDKN1A* (p21 pathway), *EGFR*, or *SNAI2* (EMT marker) did not appear to be regulated by vemurafenib.

Invasion of the epidermal cells requires proteolytic activity for degradation of, e.g., the basement membrane and dermal collagen. We, therefore, investigated also for the expression of the matrix metalloproteases *MMP1*, *MMP3*, *MMP9*, and *MMP14*, all associated with cSCCs (reviewed in 25). As shown in **Figure 2** and particularly striking, *MMP1* and *MMP3* were induced immediately and strongly in all three keratinocyte variants, suggestive of being direct targets of the MEK-ERK hyperactivation. Interestingly, induction was most prominent in NHEK (>10-fold), again pointing to their high sensitivity to vemurafenib treatment (**Figure 2A**). *MMP9* expression (~2-fold after 24 h) was restricted to the HrasA5 cells (**Figure 2C**). *MMP14* did not seem to be regulated by vemurafenib treatment in any of the keratinocytes (**Figures 2A–C**).

Together, vemurafenib contributes to keratinocyte regulation by directly inducing epidermal differentiation in NHEK and strongly upregulating the expression of components of the degradome, *MMP1* and *MMP3*, in all keratinocytes irrespective of their transformation state and genetic composition.

### Vemurafenib Improves Tissue Organization

To determine the role of vemurafenib on tissue regulation, we established skin equivalents (SEs) with NHEK, HaCaT, and HrasA5 cells. For this, dermal equivalents (DEs) were prepared by allowing the fibroblasts to establish their own dermal matrix which after 4 weeks of maturation were supplemented with the keratinocytes. By propagating the cocultures at the air-liquid interphase for 2 weeks, skin equivalents (SEs) develop that are composed of a stratified and differentiated epidermis connected to the dermal matrix through a basement membrane. Such 2-week-old SEs were then treated with vemurafenib or solvent control and histological comparison was performed after 1, 3, or 5 weeks of treatment.

**Figure 3:**
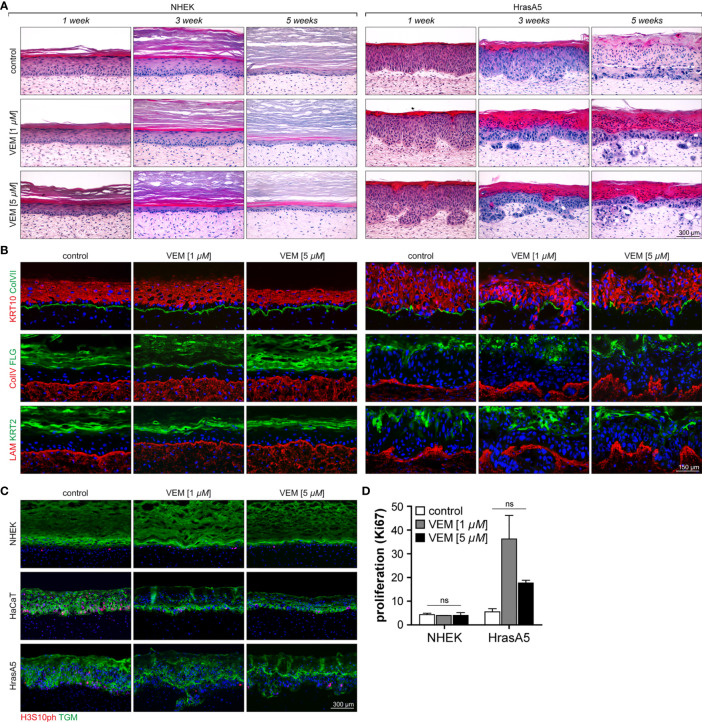
Characterization of the effects of vemurafenib on epithelial differentiation and proliferation in SEs. SEs from NHEK and HrasA5 cells were treated with vemurafenib (VEM) (1 and 5 µM) for up to 5 weeks and histology and immunostaining were performed at the indicated time points. **(A)** H&E staining of SEs from NHEK demonstrates accelerated cornification, particularly evident upon 5 µM VEM (left). Also, HrasA5 epithelia showed a time-dependent increase in cornification. In addition, invasion was seen after 3 (1 µM VEM) and 1 week (5 µM VEM), respectively (right). **(B)** Improved differentiation is confirmed by immunostaining for the early, KRT10, and the late differentiation markers FLG and KRT2 in the epithelium of the NHEKs (left) and the HrasA5 cells (right). The BM components COLVII (green), COLIV (red), and LAM (red) are expressed as contiguous lines in NHEK SEs at all time points and all conditions. Note that COLIV is expressed continuously and present throughout the DE; though enriched in the BM zone (left). In HrasA5 SEs, COLVII is generally reduced and lost at the invasive front. COLIV and LAM rather appear “bloated” with the tumor cells pushing through small gaps (right). **(C)** Same SEs stained for the proliferation marker H3S10ph (red), demonstrating very similar proliferation for all NHEK SEs (left). In HrasA5 SEs, proliferation is present throughout the epithelium (control). Under VEM, proliferation gets restricted to the basal compartment (right). All SEs are counterstained for the early differentiation marker transglutaminase 1 (TGM). **(D)** For quantification of proliferation SEs from NHEK and HrasA5 cells were costained with COLVII and KI67 and the number of proliferating cells (KI67+) correlated with BM (COLVII) length. Neither NHEKs nor HrasA5 cells showed a significant regulation in proliferation (*n* = 2, mean + SEM, one-way ANOVA + Dunnett’s multiple comparison test; ns, not significant). For all conditions, nuclei were counterstained with DAPI (blue). Treatment with the DMSO was used as control. Time specifications relate to time after starting the scale bar = 300 µm [for **(A, C)**]; scale bar = 150 µm [for **(B)**].

NHEKs start with a wound-like hyperplastic epidermis (first 4 to 5 weeks), which is reduced by reaching tissue homeostasis and long-term regeneration is maintained by an equilibrium of proliferation and differentiation (>week 5). As it is not shed, the stratum corneum continuously expands (**Figure 3A**; 20). Exposure to 1 µM vemurafenib interfered little with tissue morphology, though the epidermis appeared more compact with a slightly reduced number of cell layers and a tendency for an increased stratum corneum. Exposure to 5 µM vemurafenib, however, caused accelerated differentiation leading to significant reduction in living cell layers and hyperkeratosis after only 1 week of treatment (**Figure 3A**).

HaCaT cells form a multilayered parakeratotic epithelium that, in contrast to NHEKs, becomes atrophic upon long-term regeneration (7 weeks) with only few remaining basal cells that are unable to properly connect the epidermis with the underlying dermal equivalent (**Supplementary Figure S2A**). Upon vemurafenib treatment, the epithelium reorganized with a tendency for improved epidermal morphogenesis and differentiation as indicated by the formation of a *stratum granulosum* and an extended and regularly structured parakeratotic *stratum corneum*. Importantly, upon long-term treatment a vital epithelium was maintained, suggesting that Vemurafenib induced a differentiation-dependent tissue normalization being connected with longevity of the HaCaT cells in the *in vivo*-like environment.

Likewise, the premalignant HrasA5 cells form a hyperplastic, moderately differentiated surface epithelium with an undulated BM zone. Of note, also these epithelia become atrophic during long-term propagation (see control of **Figure 3A**). Upon vemurafenib treatment, differentiation was strongly increased, indicated by a steadily growing parakeratotic stratum corneum and occasional horn-pearls within the epithelium (**Figure 3A**). With the shift to increased differentiation, also these epithelia gained longevity. In addition, and unique for the HrasA5 cells, vemurafenib induced rapid and extended invasion. Already after 1 week of treatment with 5 µM vemurafenib, the HrasA5 cells had broken through the BM and invaded the underlying dermal matrix. With 1 µM vemurafenib, invasion was only seen after 3 weeks, arguing for a dose-dependent regulation (**Figure 3A**).

To confirm and extend the histological findings, we next analyzed the expression and distribution of the early (KRT10) and terminal epidermal differentiation markers keratin 2 (KRT2) and FLG. In NHEK SEs, KRT10 was expressed in all suprabasal layers. KRT2 and FLG, on the other hand, were increased with vemurafenib and in addition, KRT2 expression became more restricted and thus, more similar to the distribution in normal human skin (**Figure 3B**). Also, HaCaT (**Supplementary Figure S2B**) and HrasA5 epithelia (**Figure 3B**) were characterized by increased expression and more regular localization of the differentiation markers (for HrasA5 for 5 µM vemurafenib). Together, this confirms the vemurafenib-dependent advancement in epidermal differentiation and shows that an epidermal normalization also occurs to the transformed keratinocytes.

### Vemurafenib Induces Invasion in HrasA5 Cells

In addition to the induction of differentiation, vemurafenib promoted invasive growth exclusively for the HrasA5 cells. While NHEK (**Figure 3A**) and HaCaT cells (**Supplementary Figure S2A**) remained as surface epithelia throughout the 5-week treatment period with vemurafenib, HrasA5 cells invaded the underlying dermal matrix within 3 weeks when treated with 1 µM vemurafenib and even more rapidly (within the first week) when 5 µM vemurafenib was applied (**Figure 3A**).

### Vemurafenib Does Not Accelerate *In Vivo* Proliferation

In general, tumor growth correlates with increased proliferation. Although in our *in vitro* analyses vemurafenib was rather growth restrictive for NHEKs and did not seem to confer a growth advantage to the HrasA5 cells, we nevertheless determined whether in the context of invasion, vemurafenib might trigger proliferation. First, we performed staining for the proliferation marker PHH3 [phosphorylated Histone H3-H3s10ph (serine 10 phosphorylated)] on SEs treated with 1 or 5 µM vemurafenib for 3 weeks (**Figure 3C**) and found similar low proliferation for control and vemurafenib-treated NHEKs. In HaCaT SEs, vemurafenib rather reduced proliferation and mediated improved tissue organization by restricting the proliferating cells to the basal compartment. The same result was observed for the HrasA5 SEs. We additionally quantified proliferation by performing staining with the commonly established proliferation marker Ki67 (**Figure 3D**). Thereby, we found the same low proliferation rate in untreated and vemurafenib-treated NHEK SEs, suggesting that different from conventional cultures, vemurafenib does not constrain keratinocyte growth in the tissue context (see **Figure 1**). For HrasA5 cells, we find an increasing variance between different SEs, with a trend (not statistically significant) towards enhanced proliferation upon vemurafenib treatment (**Figure 3D**). Together, this suggests that the role of vemurafenib on keratinocyte growth is dependent on the respective environment, and we conclude that proliferation is not a major driving force for the invasive growth of the HrasA5 cells.

### Vemurafenib Mediates Sustained pERK Activation in SEs

To obtain more insight into the mechanism of vemurafenib-induced invasion, we first investigated vemurafenib-dependent MEK-ERK hyperactivation in the SEs. Using pERK1/2 as a marker for active ERK signaling, we show that pERK1/2 is absent in untreated epidermis but expressed in the suprabasal layers of the epidermis of vemurafenib-treated NHEK (**Supplementary Figure S3**). In HaCaT epithelia, the MEK-ERK kinase pathway appeared constitutively active as indicated by some minor pERK1/2 staining within the epithelium. Upon vemurafenib (5 µM) treatment, however, staining for pERK1/2 strongly increased throughout the living part of the epithelium (**Supplementary Figure S2C**). Similarly, in HrasA5 epithelia, pERK1/2 was already increased upon treatment with 1 µM vemurafenib, and expression became strongly upregulated with 5 µM vemurafenib where it was particularly prominent at the invasive front and in the invasive nodules (**Supplementary Figure S3**). As proof for the presence of ERK, nonphosphorylated ERK1/2 was shown to be expressed rather evenly throughout the epithelium. Thus, corresponding to the expression in conventional (2D) cultures (see **Figure 1**), vemurafenib induced and sustained hyperactivation of the MEK-ERK pathway also in the epithelia of the SEs.

### Vemurafenib Does Not Regulate Basement Membrane Integrity

To determine the conditions for invasive growth, we asked whether the basement membrane (BM) structure may be modulated by vemurafenib in order to precondition the environment for rapid tumor cell invasion. We, therefore, analyzed expression and deposition of three different BM components, i.e., collagen type IV (Col IV), collagen type VII (Col VII), and laminin 332 (LAM). This study demonstrated regular expression and continuous linear deposition between epithelium and dermal equivalent for all three BM components at all time points and both vemurafenib concentrations in the NHEK-SEs (**Figure 3B**). Similarly, HaCaT SEs showed a regular distribution of the BM components also under vemurafenib treatment (**Supplementary Figure S2B**). This was, different for HrasA5 cells (**Figure 3B**). While HrasA5 control SEs exhibited a regular distribution of the BM components, treatment with vemurafenib caused changes that were however, strictly linked to invasion of the keratinocytes. Accordingly, Col VII became degraded at the invasive front and remained discontinuous further on. Col IV and LAM were rather pushed aside by the invasive front. Thus, our data suggest that the BM is not a general target of vemurafenib, instead, it gets altered/degraded only in concert with the invasive process initiated by activating the HrasA5 keratinocytes.

### Invasion Is Regulated by Vemurafenib-Dependent Induction of Epidermal MMP1 and MMP3

**Figure 4:**
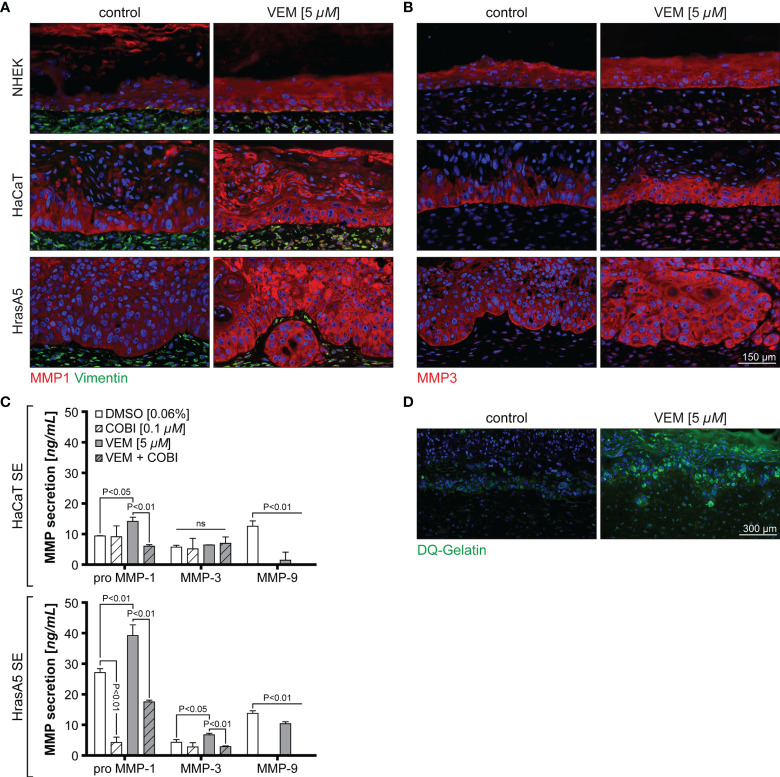
Vemurafenib-dependent degradome. SEs from NHEK, HaCaT cells, and HrasA5 cells were treated with vemurafenib (VEM) (5 µM) for 3 weeks and sections of control (DMSO) and VEM-treated SEs were stained for MMP1 and vimentin **(A)** and MMP3 **(B)**. Nuclei were counterstained with DAPI. All VEM-treated samples show a clear upregulation of both MMPs scale bar = 150 µm. **(C)** ELISA for HaCaT and HrasA5 SEs treated with DMSO (control), cobimetinib (COBI), VEM, and the combination of COBI and VEM with the indicated concentrations. Secretion of MMP1, MMP3, and MMP9 were quantified (*n* = 3, mean ± SD, one-way ANOVA + Dunnett’s multiple comparison test; ns, not significant). **(D)** Gelatinase assay confirms a strong proteolytic activity (green) in the VEM-treated (5 µM) HrasA5 SEs compared with the untreated controls. Scale bar = 300 μm.

The *in vitro* expression analyses have shown that MMP1 and MMP3 represented the genes with the strongest upregulation upon vemurafenib treatment and irrespective of the transformation state of the keratinocytes. Nevertheless, only HrasA5 cells showed BM degradation and active invasion into the underlying dermal matrix when grown in SEs. To address this discrepancy, we first investigated MMP expression in the SEs (**Figures 4A, B**). Staining of the different SEs for MMP1 and MMP3 showed that both MMPs were expressed upon vemurafenib treatment (5 µM) in all epithelia of NHEK, HaCaT, and HrasA5 cells (**Figures 4A, B**). However, and as compared with NHEK SEs, there appeared to be increased expression of MMP1 in the HaCaT and HrasA5 epithelia (**Figure 4A**) and a particular increase in the HrasA5 epithelia for MMP3 (**Figure 4B**). It is noteworthy that MMP1 and MMP3 were expressed also by the dermal fibroblasts (**Figures 4A, B**).

To determine whether the differences in staining intensity may point to quantitative differences in the expression of MMP1 and MMP3 in HrasA5 versus HaCaT SEs, we next performed ELISA for secreted MMP1 and MMP3. As MMP9 mRNA has shown a slight upregulation in the cultured cells, particularly in HrasA5 cells (see **Figure 2A**), we also included MMP9 in this analysis. While HaCaT SEs only secreted significantly increased levels of pro-MMP1, HrasA5 SEs demonstrated significant levels of both MMP1 and MMP3 upon vemurafenib treatment (5 µM) for 3 weeks (**Figure 4C**). MMP9 was present at similar levels with and without vemurafenib, suggesting that MMP9 is not induced by MEK-ERK hyperactivation and thus, not of major relevance for HrasA5 invasion in this scenario.

In line with the increased MMP secretion, proteolytic activity, as assessed by the gelatinase assay, was only seen in HrasA5 SEs (**Figure 4D**), suggesting that MMP3 either alone or in combination with MMP1 is mainly responsible for invasion of HrasA5.

### Inhibition of MMP Causes Reversion of the Invasive Phenotype

**Figure 5:**
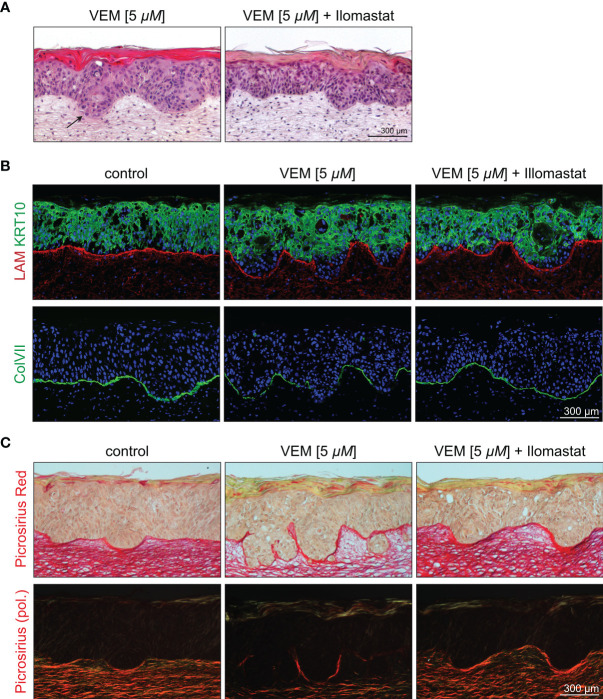
Inhibition of the vemurafenib-dependent degradome in HrasA5 SEs by suppressing MMP activity. **(A)** H&E-stained HrasA5-SEs under vemurafenib (5 µM) exhibit an invasive phenotype with signs of subepithelial disintegration under the invading pegs (left, arrow), a physiological state not seen in SEs treated in addition with the MMP inhibitor ilomastat (10 µM) (right). **(B)** Staining for the BM-components LAM (red, above) and ColVII (green, bottom) points to a pronounced degradation upon vemurafenib (middle) when compared with untreated control SEs (left). Additional application of ilomastat prevents proteolysis and allows for an uninterrupted BM (right). Likewise, delayed onset of KRT10 expression (green, middle, above) is widely renormalized by ilomastat (right, above). **(C)** Beyond the BM, also the subepithelial extracellular matrix is affected by the increased proteolytic activity. Picrosirius red staining visualizes semiquantitatively the amount of stromal collagen in bright field microscopy (above) and even more clearly in circular polarization microscopy that specifically highlights organized collagen bundles (bottom). Whereas under vemurafenib the density of collagen fibers is drastically reduced to 48.80% (middle), cotreatment with ilomastat completely preserves the control state (100.85% vs. 100%, right vs. left). Scale bar = 300 µM.

To substantiate the role of the MMPs for vemurafenib-dependent invasion, we established SEs with HrasA5 cells and selectively interfered with MMP activity by cotreatment with vemurafenib (5 µM) and the broad band MMP inhibitor ilomastat (10 µM). SEs treated with vemurafenib only, revealed the typical induction of differentiation and invasion (**Figure 5A**). Upon cotreatment with ilomastat, the vemurafenib-specific differentiation phenotype was maintained, i.e., the cells continued to build a surface epithelium with a massive stratum corneum. However, the invasive phenotype was clearly suppressed. The early phase of vemurafenib-induced invasion is characterized by a smooth borderline of the invasive front extending into the stroma (**Figure 5A**) and degradation of the BM components LAM and most extensively Col VII (**Figure 5B**). In addition, dermal collagen fibers were affected. By picrosirius red staining, that allows a quantitative morphometric evaluation of the collagen bundles under polarized light, we could confirm the degradation of collagen fibers below the epithelium and at the invasive front of vemurafenib-treated SEs (**Figure 5C**). The entire process was halted upon the addition of ilomastat as shown by a sharp demarcation zone (**Figure 5A**) with a continuous linear deposition of LAM and Col VII (**Figure 5B**) as well as preservation of the collagen fiber network (**Figure 5C**). A confirmative quantification of fibrillar collagen was performed by means of image analysis of those picrosirius red-stained sections and normalized to the level of control SEs (100%, standard deviation (SD) 18.80%). Vemurafenib-treated SEs showed a reduction to 48.80% (SD 13.18%) that was fully outweighed to 100.85% (SD 17.04%) by cotreatment with ilomastat. Together, these data support our hypothesis that vemurafenib-dependent induction of MMP1 and MMP3 is in charge of the rapid tumor cell invasion seen in SEs with the HrasA5 cells.

### MEK Inhibitor Cobimetinib Abrogates the MEK-ERK Hyperactivation of Vemurafenib

It is suggested that combination therapy of vemurafenib with the MEK inhibitor cobimetinib is not only more effective in combating resistance of melanoma cells but also in preventing cutaneous adverse events, including the formation of cSCCs [for review, see, e.g., (26)]. Cobimetinib is inhibiting MEK1 (and partially also MEK2) and thereby hinders ERK1/2 phosphorylation and abrogates vemurafenib-dependent MEK-ERK hyperactivation (27). Therefore, we asked how cobimetinib would affect epithelial tissue regulation and whether it would be able to counteract the vemurafenib-induced cutaneous adverse events.

As the RAS-MEK-ERK pathway is involved in many important cellular functions, we were concerned about possible toxicity when completely blocking MEK function in cells or tissues. We therefore first applied 0.1 and 1 µM cobimetinib alone for 3 weeks to HrasA5 SEs. While 0.1 µM cobimetinib already caused some tissue atrophy, strong toxicity was seen with 1 µM, as only remnants of epithelial islands remained, and the number of dermal fibroblasts was reduced as well (**Supplementary Figure S4A**).

We next sought for the cobimetinib concentration which would be sufficient to counteract vemurafenib-induced MEK-ERK hyperactivation by establishing a dose-response profile for the HrasA5 cells. Cultures of HrasA5 cells were treated with either 1 or 5 µM vemuarafenib in combination with increasing doses of cobimetinib (0.01, 0.1, and 1 µM). We found a concentration-dependent increase in P-MEK—due to inhibition of active, phosphorylated MEK [e.g., (12)]—and a blockade of ERK1/2 phosphorylation because of inhibited MEK activity (**Supplementary Figure S4B**). As little as 0.01 µM cobimetinib was sufficient to block ERK1/2 phosphorylation in combination with 1 µM vemurafenib, while 0.1 µM was required to block ERK1/2 phosphorylation induced by 5 µM vemurafenib. Thus, by combining the data from the toxicity assay with the inhibitory capacity of the vemurafenib-induced hyperactivation by cobimetinib, 0.1 µM was chosen as concentration for further assays.

To determine the molecular consequence accompanying abrogation of ERK signaling, we treated HrasA5 cells with vemurafenib (5 µM) alone or in combination with cobimetinib (0.1 µM) and analyzed transcription of those genes that were regulated by vemurafenib (**Supplementary Figure S4C**; see **Figure 2A**). Accordingly, expression of *TGF-α*, *IL-1A*, *IL-1B*, *IL-8*, *MMP1*, *MMP3*, and *MMP9*, was inhibited upon cotreatment with cobimetinib. *MMP-14* remained unaffected and unexpectedly, the differentiation-specific genes, *KRT10* and *FLG*, were stimulated upon cotreatment, corroborating that the epidermal differentiation markers are not regulated directly by MEK-ERK signaling in the transformed HrasA5 cells.

### Cobimetinib Cotreatment Impedes Vemurafenib-Induced Differentiation and Invasive Growth

To determine the physiological relevance of vemurafenib-cobimetinib cotreatment, we next performed SEs with HrasA5 cells treated with vemurafenib (5 µM) or cobimetinib (0.1 µM) applied for 3 weeks either individually or in combination and investigated for their functional consequences.

**Figure 6:**
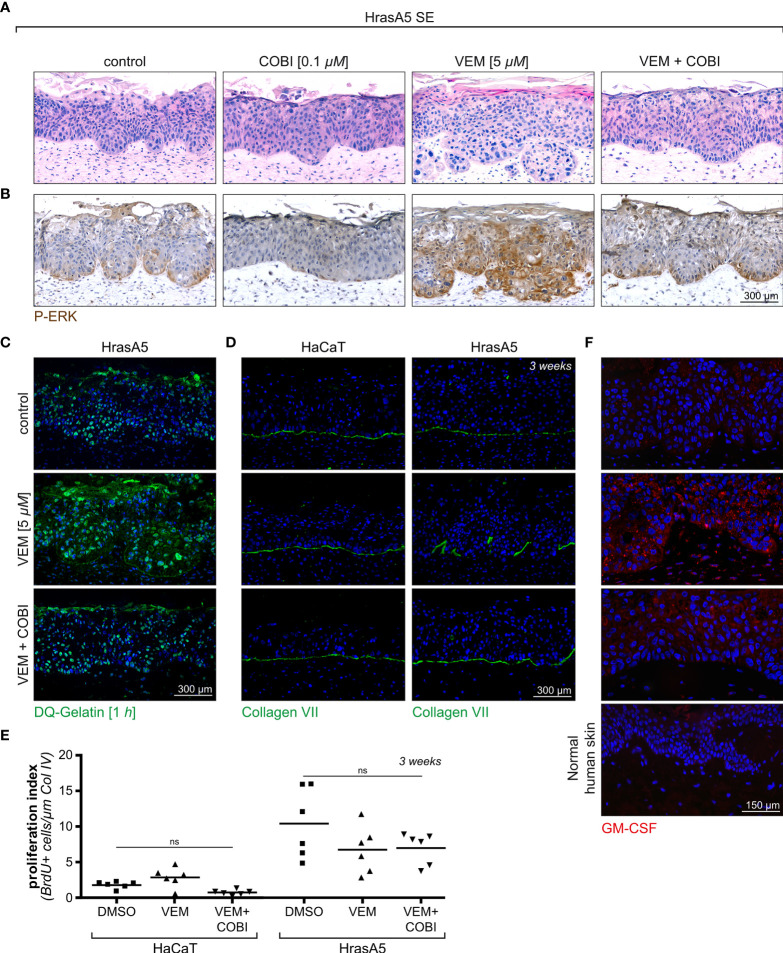
Cotreatment of HrasA5-SEs with vemurafenib and cobimetinib. **(A)** HrasA5 SEs were treated with vemurafenib (VEM) alone (5 µM), cobimetinib (COBI) (0.1 µM) alone, or the combination of both inhibitors for 3 weeks. The histologic H&E stainings demonstrate the respective phenotypes. **(B)** Immunohistochemistry for pERK1/2 of corresponding sections confirms the vemurafenib-dependent increase in pERK and its suppression down to control level by cobimetinib. **(C)** Proteolytic activity *in situ*, visualized by the fluorescent gelatinase assay, results in an intense labeling in vemurafenib-treated SEs (middle) as opposed to a weak labeling in vemurafenib + cobimetinib-treated SEs (bottom), thus providing functional proof for the drop in MMP-expression caused by cobimetinib. **(D)** Immunostaining for COLVII (green) as indicator for BM integrity demonstrates the effectivity of cobimetinib in blocking degradation and invasion in HrasA5-SEs (right column). The noninvasive HaCaT-SEs have an undisturbed BM under all conditions tested (left column). **(E)** Quantification of proliferating cells in whole mounts of HaCaT- and HrasA5-SEs (BrDU positive cells/µm of Col IV) does not show significantly diverse proliferation rates under the different conditions (*n* = 3, mean, one-way ANOVA + Dunnett’s multiple comparison test; ns, not significant). **(F)** GM-CSF is only faintly detectable by immunofluorescence analysis (red) in normal skin (4th from top) and in HrasA5-SE controls (top) while clearly visible in the epithelium and dermal fibroblasts of vemurafenib-treated SEs (2nd from top). Cotreatment with cobimetinib largely abrogates the GM-CSF signals (3rd from top). Scale bar = 300 µm [for **(A–D)**] Scale bar = 150 µm [for **(F)**].

As demonstrated in **Figure 6**, the treatment of HrasA5 SEs with cobimetinib (0.1 µM) largely abolished pERK1/2 expression, and as expected from the previous experiments (see **Supplementary Figure S4**), this correlated with signs of epithelial atrophy. Vemurafenib (5 µM), on the other hand, caused the characteristic MEK-ERK hyperactivation, as indicated by strong and extended pERK1/2 staining, particularly also at the invading front. This increased ERK activation correlated with pronounced differentiation, recognized by a distinct parakeratotic stratum corneum, and with rapid and ongoing tumor cell invasion. Importantly, cotreatment with cobimetinib strongly reduced the level of pERK1/2, and this was sufficient to hinder atrophy but also to impede both, induction of accelerated differentiation and tumor cell invasion. It is also important to note, that pERK1/2 was not completely abolished but reduced to a similar level as in controls, likely owing to the active Hras oncogene, what might be the reason that epithelial atrophy was counteracted (**Figure 6A**). Repeating a similar set of experiments with the HaCaT cells demonstrated an even more pronounced inhibition of pERK 1/2 upon treatment with cobimetinib alone or in combination with vemurafenib, with the consequence of increased epithelial atrophy (data not shown).

With the halted expression of the MMP RNAs upon cotreatment of vemurafenib and cobimetinib (see **Supplementary Figure S4**) also secretion of the MMPs was inhibited as quantified by enzyme-linked immunosorbent assay (ELISA) (see **Figure 4C**). As a consequence of this, proteolytic activity was strongly reduced in the epithelia of the HrasA5 SEs (**Figure 6C**) and BM integrity was maintained, as demonstrated for the most critical BM component, COL VII, in both the HaCaT and HrasA5 SEs (**Figure 6D**).

Finally, we labeled untreated, vemurafenib-treated, and vemurafenib+cobimetinib cotreated SEs of HaCaT and HrasA5 cells with BrdU to detect S-phase cells in epidermal whole mounts (28). As epidermal proliferation is generally focal, we used this third and most unbiased approach for ensuring precise enumeration (**Figure 6E**). Also, the analysis of large tissue areas demonstrated that vemurafenib did not induce proliferation, particularly not in HrasA5 epithelia, and that the combination treatment did not affect proliferation as well.

Together, this indicates that cobimetinib efficiently blocks the vemurafenib-dependent MEK-ERK hyperactivation. It thereby clearly demonstrates that both, the vemurafenib-specific differentiation and the invasion phenotype, are under direct control of MEK-ERK hyperactivation. Furthermore, our multistep skin carcinogenesis model revealed the ras-oncogene as decisive element provoking immediate tumor cell invasion.

### Vemurafenib Also Modulates the Microenvironment: Vemurafenib Causes a Differential Expression Profile in Dermal Fibroblasts in Conventional Cultures Versus Dermal Equivalents

**Figure 7:**
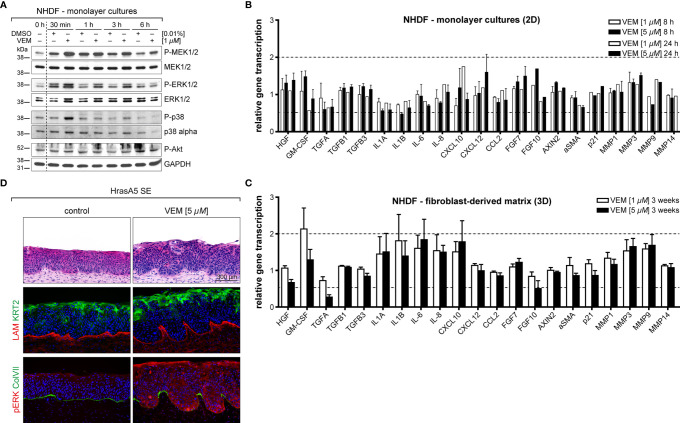
The impact on vemurafenib (VEM) the dermal microenvironment. **(A)** Western blot analysis of vemurafenib-treated fibroblasts (1 µM in monolayer cultures) demonstrates a quick rise in pMEK, pERK, and pp38 alluding to a pathway activation. **(B)** RNA expression analysis of fibroblast monolayer cultures for genes that are relevant for epidermal-stromal interaction demonstrates only little gene regulation upon vemurafenib. **(C)** RNA expression analysis for the same genes in 3D-cultivated fibroblasts (DEs), treated for 3 weeks with vemurafenib, shows enhanced expression of GM-CSF, interleukins, and MMPs. For **(B, C)**, normalization was performed using GAPDH as a house-keeping gene and foldchanges were expressed by comparing 1 or 5 µM vemurafenib treatment of NHDF to DMSO stimulation. *n* = 2, mean ± SD. **(D)** Vemurafenib-pretreated DEs are able to convey the vemurafenib effect when combined with HrasA5 keratinocytes: H&E stainings of HrasA5-SEs demonstrate invasion and differentiation of HrasA5 cells (top, right) as compared with untreated controls (top, left). Immunofluorescence staining for COL IV (red) presents a regular continuous BM in controls (middle left) while being reduced under the invading epithelial pegs on pretreated DEs (middle right). In the latter, boosted differentiation is indicated by enhanced KRT2 (green). In control SEs, pERK is not detectable (red, bottom left), instead a continuous BM is demarcated by COL VII (green). On pretreated DEs, HrasA5 epithelia show intense pERK staining, being particularly enriched in the invading epithelial pegs. COL VII is degraded, leaving only small patches between the invading fronts (bottom, right). Scale bar = 300 µm.

As tissue regulation is controlled by an extensive communication and interaction between epidermis and dermis, we hypothesized that vemurafenib may similarly affect the microenvironment, i.e., the dermal fibroblasts. To address this, we first determined whether normal human dermal fibroblasts (NHDFs) are activated by vemurafenib in a similar manner as the human skin keratinocytes. Two fibroblast strains derived from adult trunk skin were treated with 1 and 5 µM vemurafenib in conventional culture (**Figure 7A**). This caused sustained activation of pMEK and pERK and temporary activation of p38 (at 30 min), demonstrating that vemurafenib-dependent MEK-ERK hyperactivation also arises in the BRAF wildtype dermal fibroblasts.

We next established an expression profile for different growth factors, cytokines, MMPs, indicators for the Wnt (*AXIN2*) and p21 (*CDKN1A*) pathway, and myofibroblast transformation [alpha smooth muscle actin (*αSMA*)] (**Figure 7B**). Different from the distinct regulatory activity seen in the keratinocytes, only little gene regulation occurred in the fibroblasts and none of the selected genes reached 2-fold regulation. *CXCL12*, *CXCL10*, *FGF7 (KGF)*, *FGF10*, and *CSF2* (GM-CSF) as well as *MMP3* and *MMP9* were upregulated approximately 1.5-fold.

In conventional cultures, fibroblasts are permanently activated and highly proliferative while *in situ*, i.e., in intact skin or in DEs, they are largely growth arrested. To determine how this physiological difference would influence the expression response to vemurafenib, we investigated the expression of the same genes in fibroblasts from DEs. To exclude any epidermal impact, “naked” DEs without keratinocytes were treated with vemurafenib (1 and 5 µM) for 3 weeks and further analyzed. Interestingly, we saw an increase in the number and intensity of genes regulated by vemurafenib. Specifically, we found a >1.5-fold induction of *IL-1A* and *IL-1B* (both also regulated in the keratinocytes), *IL-6*, *IL-8*, and *CXCL10*. *CSF2* (GM-CSF), an important regulator of epidermal growth and differentiation (29, 30) even reached a 2-fold increase. *MMP3* and *MMP9* were upregulated (1.75-fold) and to a very minor degree also *MMP1*. *MMP14* remained unaffected also in the fibroblasts, pointing again to a specific set of MMPs as being direct targets of MEK-ERK hyperactivation (**Figure 7C**).

### Vemurafenib-Pretreated DEs Contribute to the Vemurafenib-Specific Epidermal Phenotype

To determine whether and how this regulation might contribute to the vemurafenib-dependent skin phenotype, we added HrasA5 cells onto vemurafenib-pretreated DEs and maintained them as cocultures (SEs) for 3 weeks without further addition of vemurafenib. Most excitingly, histology of these SEs indicated that an epidermal phenotype was established (**Figure 7D**). Vemurafenib-dependent preconditioning of the DEs contributed to epidermal differentiation, as demonstrated by an increase in KRT2-positive cells and the appearance of horn-pearls within the epithelium. In addition, the keratinocytes formed large protrusions advancing into the dermal equivalent. While LAM was still present as a contiguous line, though strongly diminished at the invasive sites, Col VII was largely absent at the basal pole of the protrusions (**Figure 7D**), suggesting an incipient BM degradation. Staining for pERK1/2 clearly demonstrated ERK activation in the dermal fibroblasts (**Figure 7D**). Interestingly, pERK1/2 was also evident in the epidermal protrusions. Although some pretreatment-dependent storage of vemurafenib cannot be excluded, it is tempting to suggest this is the result of a paracrine ERK activation through the vemurafenib-stimulated microenvironment.

Based on the fact that *CSF2* (GM-CSF) was the most upregulated gene in the vemurafenib-treated DEs, we hypothesized that the increase in GM-CSF would contribute to the epidermal phenotype, i.e., would support accelerated epidermal differentiation upon vemurafenib treatment. Accordingly, we show a strong increase in GM-CSF in vemurafenib-treated SEs (**Figure 6F**) and demonstrate that cotreatment with cobimetinib abolished the overexpression of GM-CSF (**Figure 6F**) as it prevented increased differentiation.

Together, these data demonstrate that dermal fibroblasts are also activated by vemurafenib and contribute with their specific expression profile to the epidermal phenotype. This highlights the importance of the interplay of epidermal and dermal regulation on the skin phenotype upon vemurafenib treatment.

### Perilesional Skin and cSCCs of Vemurafenib-Treated Melanoma Patients Exhibit a Similar Expression Profile as SEs

Finally, to establish the connection of our experimental findings to clinical cases, we investigated biopsies from vemurafenib-treated melanoma patients. Samples of perilesional skin and different skin tumors (viral warts and different stages of cSCC) were analyzed for hyperactivation of the MEK-ERK pathway by staining for pERK1/2, activation of epidermal MMP1 and MMP3 expression, as well as stromal upregulation of GM-CSF.

**Figure 8:**
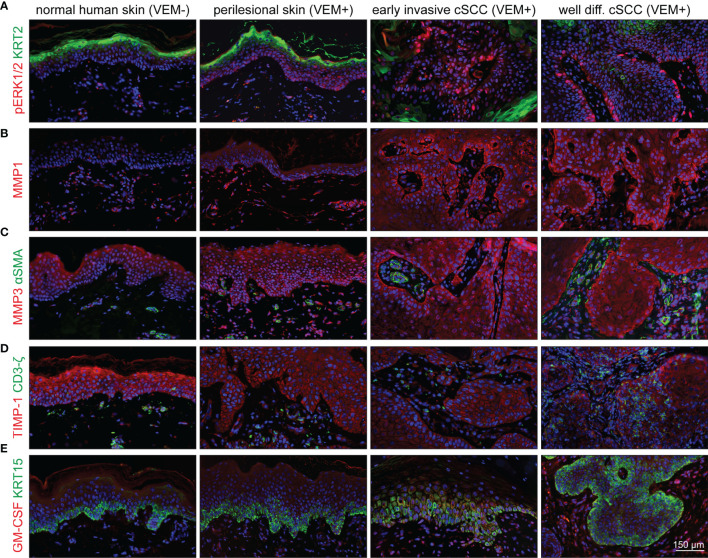
Activation of the MEK-ERK pathway in cutaneous lesions of patients under vemurafenib treatment. Components and targets of the pathway are detected by immunofluorescence microscopy in normal skin (first column) compared with perilesional skin (second column), early invasive SCCs (third column), and well-differentiated SCCs (fourth column). **(A)** Under vemurafenib, pERK (red) is upregulated in the nuclei of basal epidermal cells; KRT2 (green) demarcates terminally differentiated upper epidermal cells. **(B)** The intensity of MMP1 staining (red) significantly increases under vemurafenib treatment and shows enrichment in the invading epithelia. **(C)** MMP3 displays a moderate epithelial staining (red) in normal skin that gains intensity under treatment and progression towards SCC. Also dermal cells stain positive for MMP3. No signs of myofibroblast differentiation; αSMA (green) staining is restricted to vascular structures. **(D)** Decreased intensity of TIMP-1 (red) correlates with an acquired invasive phenotype under vemurafenib; CD3ζ-positive T-cells (green) do not show any significant accumulation. **(E)** Faint epithelial GM-CSF signals (red) are accompanied by a well-detectable staining of stromal cells under vemurafenib, particularly in SCCs. The epidermal basal layer is defined by staining for KRT15 (green). Scale bar = 150 µm.

Comparing normal skin from healthy donors to perilesional skin from the melanoma patients, we found a slight increase of pERK1/2 as well as an even more extensive increase in pERK1/2 expression in warts and tumor samples (**Figure 8A**), thereby confirming vemurafenib-dependent MEK-ERK activation also in skin keratinocytes *in situ*. Similarly, we found expression of MMP1 and MMP3 with a rise in staining intensity from perilesional skin to cSCC (**Figures 8B, C**). As staining for both MMPs was particularly prominent in the epithelium, it is tempting to suggested that the keratinocytes are the primary target for the vemurafenib-stimulus inducing MMP expression. In addition, staining for the tissue inhibitor of metalloproteinases, TIMP-1, as one of the endogenous MMP inhibitors, showed reduction in perilesional skin as well as tumor samples as compared with normal healthy skin (**Figure 8D**), which may support an increasing imbalance in favor of the degradative phenotype with vemurafenib treatment. GM-CSF was hardly detected in normal human skin but expressed by fibroblasts of perilesional skin and strongly enhanced in the fibroblasts of the tumor microenvironment (**Figure 8E**), suggestive of a role for GM-CSF in the paracrine regulation of epidermal growth and differentiation also *in situ*.

Activated fibroblasts, such as cancer-associated fibroblasts (CAFs), are believed to have a strong tumor-modulating potential also for cSCC (31, 32). In agreement with our experimental analysis which did not provide evidence for a vemurafenib-dependent regulation of *TGF-β* or *αSMA* in fibroblasts (see **Figure 7C**), we found αSMA-positive blood vessels for all tissue samples but rarely identified αSMA-positive fibroblasts suggesting that myofibroblasts/CAFs may not be a major player in vemurafenib-induced cSCC development (**Figure 8C**).

In recent years, the role of immune cells in controlling tumor growth and progression has also gained considerable interest. Accordingly, immunosuppressive regulatory T cells (Tregs) were shown to be present in the immune infiltrate of cSCCs, and it was suggested that they may contribute to an ineffective antitumor immune response and promote SCC development, aggressiveness, and metastasis (33, 34). Utilizing a CD3ζ antibody, we stained all tumor samples for the T-cell receptor. Being aware of the fact that CD3ζ is not all-embracing for immunoregulatory T cells, our stainings indicate that with the exception of one tumor, showing some accumulation of T cells in the stroma and within the tumor, all others only showed few T cells scattered throughout the stroma (**Figure 8D**).

Taken together, we find a similar regulation in perilesional skin and cSCCs of the vemurafenib-treated melanoma patients as we did in our skin and skin cancer models. This directly links vemurafenib-induced MEK-ERK hyperactivation to increased differentiation and hyperkeratotic lesions, as well as to MMP-dependent tumor cell invasion, which culminates in rapid onset of cSCCs. In addition, we demonstrate a role for a tight interaction of the epidermal keratinocytes and the stromal fibroblasts in the development of the vemurafenib-specific phenotype while carcinoma-associated fibroblasts (CAFs) and/or regulatory T cells may not be of major relevance for this process.

## Discussion

In this study, we demonstrate that vemurafenib hyperactivates MEK-ERK signaling in two major cell types of the skin, the epidermal keratinocytes and the dermal fibroblasts, and highlight the importance of this activation for the underlying pathogenesis. We utilized long-term skin equivalents to allow for the investigation of both cell types in their physiological environment. In addition, we used keratinocytes of different genetic background (NHEK, HaCaT [p53 mut], and HrasA5 [p53 mut + Hras mut]. Thereby, we confirm vemurafenib-dependent hyperactivation of the MEK-ERK pathway in the epithelia of all keratinocyte variants and confirmed a similar regulation as seen in monolayer cultures of dermal fibroblasts and keratinocytes. These findings are in line with the observed hyperactivation in several BRAF wildtype cell lines that can be explained by dimerization and downstream activation of RAF proteins due to sterical properties of vemurafenib (12, 13, 35). Accordingly, Poulikakos et al. (35) had shown that binding of vemurafenib to the ATP-binding site of one kinase of the RAF dimers (either as CRAF homodimers or CRAF-BRAF heterodimers) causes transactivation of the drug-free protomer and that this transactivation was required for hyperactivating ERK signaling. Thereby, they demonstrated that inhibitor-dependent sterical transactivation was the reason for the paradoxical activation of the CRAF enzyme (35).

Alternatively, MAP3K8, the gene encoding COT/Tpl2, was identified as a MAPK pathway agonist that activates ERK primarily through MEK-dependent mechanisms that do not require RAF signaling (36). Furthermore, TPL2 downstream signaling mediated cell transformation in immortalized human keratinocytes and tumorigenesis in mice and was shown to be overexpressed in metastatic cSCC and KAs (37), Vemurafenib-dependent ERK activation *via* release and activation of COT/TPL2 may also be considered for the human keratinocytes.

Regardless of pathway initiation, we show that vemurafenib-dependent MEK-ERK hyperactivation is associated with accelerated differentiation and hyperkeratosis in the normal keratinocytes and normalized stratification and cornification in the transformed keratinocytes. Vemurafenib-dependent pathophysiological changes, i.e., the initiation of invasive growth, as part of tumorigenic conversion, is exclusive for the ras oncogene-expressing keratinocytes, suggesting a combined mechanism of MEK-ERK activation and the intersecting ras oncogenic network. This is also in line with investigations on the mode of action by vemurafenib in BRAF wildtype cells, suggesting the requirement for an upstream Ras activity for the induction of pathway hyperactivation (12).

This interpretation is based on the fact that neither HaCaT cells with vemurafenib-induced hyperactivation of MEK-ERK signaling nor Hras oncogene expressing cells with intrinsic RAS-MEK-ERK activation gain a tumorigenic phenotype. Only the combination of vemurafenib-induced MEK-ERK hyperativation with Hras oncogene expression in the HrasA5 cells, leads to the rapid onset of a proteolytic phenotype with secretion and activation of MMP1 and MMP3. This prepares the path for the keratinocytes to degrade and penetrate the BM and to invade into the degraded underlying collagen matrix. Thus, the MEK-ERK/MMP axis represents the most important molecular basis for the switch towards tumorigenic conversion upon vemurafenib treatment.

The MEK-ERK-signaling pathway as an integral part of the cellular regulatory network is classically induced through epidermal growth factor receptor (EGFR) or constitutively active in ras oncogene-containing cells and is involved in cell proliferation, migration, and inhibition of apoptosis (16). In agreement with previous studies, we show that the BRAF inhibitor vemurafenib causes MEK-ERK hyperactivation in human skin keratinocytes and dermal fibroblasts, and thereby induces a variety of physiological consequences. Accordingly, it was shown earlier, that vemurafenib stimulated growth in HaCaT cells (18) and mouse skin (14). Interestingly, for two different normal keratinocyte strains (NHEK) as well as the p53-mutant HaCaT cells and its ras-containing variant HrasA5, we could not confirm vemurafenib-dependent growth stimulation. On the contrary, NHEK (2D cultures) were growth inhibited when exposed to 1 and 5 µM vemurafenib and HaCaT cells showed delayed growth reduction upon 5 µM vemurafenib. However, this *in vitro* growth response is not maintained under *in vivo*-like conditions in the skin equivalents. When we analyzed cellular proliferation by three different methods, we realized that vemurafenib neither exerts a reduction nor a distinct increase in cell growth, and so, did not provide a significant growth advantage for any of the keratinocytes. Thus, we must conclude, that control of proliferation is not a major factor driving vemurafenib-dependent pathogenesis in our culture models.

Instead, we show that the most obvious and reproducible physiological consequence of vemurafenib is the induction of epidermal differentiation, correlating well with the clinical picture of hyperkeratotic lesions frequently encountered as adverse events in vemurafenib-treated patients with melanoma (38). It is noteworthy that the sensitivity for induction of differentiation appears reduced in the transformed keratinocytes. While NHEK show vemurafenib-dependent expression of several epidermal differentiation markers (*KRT10*, *INV*, *FLG*), increased expression of these genes was not seen by short-term stimulation of HaCaT or HrasA5 cells, but rather upon continuous treatment of HaCaT cells with 5 µm vemurafenib for 4–8 weeks (*KRT10* and *FLG*). Whether this points to an indirect regulation, remains to be seen.

Induction of differentiation was most obvious in NHEK when grown as SEs. At the expense of a hyperplastic epidermis (as in controls), accelerated cornification resulted in the formation of a massive stratum corneum and reduction of the number of vital cell layers especially within the stratum spinosum. Induction of differentiation was also conspicuous for the transformed keratinocytes (HaCaT and HrasA5) in the *in vivo*-like SEs, with a similar consequence of a reduced vital epithelium with increased and more normally distributed epidermal differentiation markers and an expanded parakeratotic stratum corneum. Together, this suggests that induction of epidermal differentiation is an unequivocal part of the action profile of vemurafenib in the human skin keratinocyte. In good agreement with this is the high frequency of hyperkeratoticlesions in vemurafenib-treated melanoma patients, as well as the well-differentiated and keratoacanthoma-type cSCC as the most prevalent skin carcinoma type in these patients (3, 39).

In addition to the activation of the keratinocytes, we also identified vemurafenib-dependent activation in the dermal fibroblasts and propose a distinct role for the microenvironment in the vemurafenib-dependent scenario. As previously described as a double paracrine loop (23, 24), epidermal IL-1A and IL-1B act on the dermal fibroblasts by inducing the expression of KGF (FGF7) and GM-CSF (CSF2), which in turn act on the keratinocytes to stimulate growth, and in the case of GM-CSF, support epidermal differentiation. First, we not only find a vemurafenib-dependent increase in the expression of *IL-1A* and *IL-1B* in monolayer-cultured keratinocytes but we also find a vemurafenib-specific increase in the expression of GM-CSF in dermal equivalents, i.e., without keratinocyte interaction. In agreement with that, we find expression of GM-CSF also in the stromal fibroblasts of perilesional skin and in the microenvironment of cSCC from vemurafenib-treated melanoma patients, suggesting that the fibroblast-derived GM-CSF supports keratinocyte differentiation also *in situ.* Therefore, we hypothesize that vemurafenib-dependent upregulation of GM-CSF is a further activity contributing to the formation of hyperkeratotic lesions and well-differentiated KAs and cSCCs in the melanoma patients (40–42).

Another relevant pathophysiological consequence of vemurafenib-treatment is the rapid transition from a surface epithelium to an invasively growing tumor epithelium. To determine the genetic requirements for the vemurafenib-dependent tumorigenic switch, we utilized keratinocytes with different genetic make-up, i.e., wildtype cells (NHEK), cells with UV-indicative p53 mutations, and cSCC-characteristic chromosomal aberrations (HaCaT), as well as variants of the HaCaT cells with an additional Hras oncogene (HrasA5). Despite the induction of similar MEK-ERK hyperactivation and gene expression of a similar degradome, only the Hras oncogene-carrying variant became invasive upon vemurafenib treatment. Hras by itself is not a dominant tumor driver in the keratinocyte model. We had shown previously that introduction of Hras oncogene into HaCaT cells resulted in a variety of clones with different pathogenic phenotype from nontumorigenic to malignant tumorigenic clones (15). As the level of ras oncogene was similar in benign and malignant tumorigenic clones this is unlikely to be a determinant of the pathogenic phenotype on its own. However, in combination with vemurafenib-induced MEK-ERK hyperactivation, invasion was induced almost immediately. Besides activating the MAPK pathway, Hras can also boost signaling pathways such as the PIK3CA-AKT-mTOR axis (for review, see 43). However, our Western blot analyses have not pointed to a differential regulation in the Hras-containing versus non-ras oncogene-containing cells.

Instead, we see very similar transcriptional regulation of a selected gene panel in NHEK, HaCaT, and HrasA5 cells with vemurafenib. Particularly striking is the strong and almost identical regulation of members of the degradome. In all three keratinocyte types, *MMP1* and *MMP3* were rapidly induced, while *MMP9* and *MMP14* remained largely unaffected. Immunostaining further verified expression of MMP1 and MMP3 in all types of epithelia in the *in vivo-*like SEs. Appreciable levels of secreted pro-MMP1 and MMP3 were however only detected in HrasA5 SEs while in HaCaT SEs only the secreted pro-MMP1 level was increased. In agreement with that, proteolytic activity was restricted to HrasA5 SEs as was degradation of the BM components Col IV, Col VII, laminin, and the underlying dermal collagens. It was suggested that vemurafenib increased MMP-2 and MMP-9 in HaCaT cells (18). Our expression analysis did not confirm this but identified MMP-1 and MMP-3 as major targets of MEK-ERK activation. While MMP1 can digest Col I and III, as well as Col VII, MMP3 is supposed to digest laminin and Col IV, as well as Col III. In addition, MMP3 is essential for the activation of pro-MMP1 (for review, see 44, 45). This prompts us to hypothesize that MMP1 and MMP3 together pave the way for HrasA5 keratinocytes permitting them to invade. Accordingly, when inhibiting MMP activity by cotreatment of HrasA5 SEs with vemurafenib and the MMP inhibitor ilomastat, proteolytic activity was prohibited, BM components and dermal collagen remained intact, and invasion was prevented, thus, revealing the causal relationship of the rapid tumorigenic conversion to vemurafenib-dependent epidermal MMP1 and MMP3 expression.

Acquired resistance to vemurafenib monotherapy due to reactivation of the MAPK pathway occurs in about two-thirds of the melanoma patients (27, 46 and references therein), opening the way for new treatment modalities. Combined inhibition of MEK and BRAF V600E turned out as a successful strategy for melanoma patients and in addition, reduce the cutaneous adverse events of developing cSCCs very efficiently (47). Cotreatment of HaCaT and HrasA5 SEs with vemurafenib (5 µM) and cobimetinib (0.1 µM) prevented P-ERK1/2 expression in HaCaT epithelia, resulting in increasing atrophy. In HrasA5 SEs, P-ERK1/2 level was reduced to that of untreated controls, and this was sufficient to hinder the vemurafenib-induced accelerated differentiation, activation of the degradome, and accordingly induction of the tumorigenic switch. In particular, secretion of MMP1, MMP3, and MMP9 levels was significantly reduced by HrasA5 keratinocytes and the invasive phenotype was prevented upon addition of cobimetinib to vemurafenib treatment, thus, once more highlighting that the invasion of HrasA5 is a direct effect of MEK-ERK hyperactivation.

In essence, we here show that the primarily unexpected side effect of Vemurafenib, namely the induction of MEK-ERK, is a strong molecular driver for those phenotypic changes leading to adverse events such as hyperkeratotic lesions, KAs, and cSCC. However, for rapid tumor conversion cofactors are required such as the ras oncogene that prime the keratinocytes to become susceptible for the accelerated tumorigenesis that is fueled by vemurafenib. While the ras oncogene seems to account for not more than 30% to 60% of the cSCCs in melanoma patients (41, 48–50) also other genetic factors should be involved in keratinocyte susceptibility to vemurafenib-dependent tumorigenic conversion. Accordingly, a role for the human papilloma virus or polyomavirus is discussed (51, 52), as is the inactivation of the TGF-β pathway by TGFβR mutations (53). In addition, our genetic analysis with the HaCaT cells treated with vemurafenib for several weeks provokes the idea that the cMYC oncogene may be involved in the vemurafenib-triggered events. Of note, we hardly found new chromosomal aberrations, making vemurafenib-dependent genomic toxicity largely neglectable. Instead, the data point to a selective shift in the cell population that is promoted by vemurafenib and provides an advantage for preexisting subpopulations that, in the case of HaCaT cells, contain increased copy numbers of cMYC. Thus, further analysis is required to establish this connection and leaving the field open for the search for additional factors, other than the ras oncogene, that contribute in the establishment of vemurafenib-induced KAs and cSCCs.

## Material and Methods

### Tissue Samples

Formalin-fixed tissue sections from 13 biopsies from 9 different patients were contributed by Catherine Harwood, Centre for Cell Biology and Cutaneous Research, Blizard Institute, Barts and the London School of Medicine and Dentistry, Queen Mary University of London, London, UK. These included 1 perilesional skin, 2 viral warts, 2 benign acanthomas, and 8 cSCC. The patient material was part of a study described by Purdie et al. (52). This study was carried out in accordance with the recommendations of East London and City Health Authority local ethics committee. The protocol was approved by the East London and City Health Authority local ethics committee. All subjects gave written informed consent in accordance with the Declaration of Helsinki.

For each of the proteins of interest, up to 6 different specimens were analyzed by immunofluorescence.

### Cell Culture

NHEK and NHDF were isolated as described previously (54). NHEK, NHDF, HaCaT, HrasA5, and HaCat-rasII4 (HrasII4) were propagated as previously described (20). NHEK were grown in DermaLife K medium, complete; HaCaT HrasA5, HrasII4, and A375 were cultivated in DMEM plus 10% FCS and 1% (v/v) Penicillin/ Streptomycin at 37°C, 5% CO_2_, and 20% O_2_. Human dermal fibroblasts were cultivated in DMEM plus 10% FCS and 1% (v/v) Penicillin/ Streptomycin at 37°C, 5% CO_2_, and 5% O_2_. Mycoplasma and virus contamination was excluded for all cell types by the Multiplex Cell Contamination Test (Multiplexion, Heidelberg, Germany), and HaCaT cells and its variants were authenticated by short tandem repeat (STR) profiling (CLS, Heidelberg, Germany).

### Generation of Fibroblast-Derived Matrix-Based Skin Equivalents

Skin equivalents (SEs) were established as described in detail previously (20). In brief, NHDF cells were seeded onto a filter insert at 2-day intervals in three successive steps. During submerged cultivation for 4 weeks, the fibroblasts develop an ECM-rich dermal equivalent (DE). Keratinocytes were seeded onto the DE and after 1 day of submersed growth were cultivated at the air-liquid interface. During a cultivation period of 2 weeks, the keratinocytes develop a stratified and differentiated epithelium and thereafter, the SEs were treated with vemurafenib, vemurafenib plus ilomastat, cobimetinib, or vemurafenib plus cobimetinib, with concentrations indicated in the different experiments. In all experiments, the control cultures were treated with DMSO in a concentration used as solvent for vemurafenib and cobimetinib.

### Proliferation Assay

#### SYBR Green Proliferation Assay

Cells were seeded in 24-well plates at a density of 10^4^ cells in 500 µl medium (NHEK: DermaLife K medium complete/HaCaT and HrasA5 cells: DMEM plus 10% FCS) and incubated for 24 h before treatment. To determine the total cell number per well, plates were incubated with SYBR Green (1:2,500, Life Technologies, Carlsbad, CA, USA) in 0.1% (*v*/*v*) Triton X-100 diluted in PBS for 1 h. Fluorescence was measured at 485 nm with Fluoroskan Ascent (Thermo Fischer Scientific, Waltham, MA, USA) and fluorescence intensity correlated to a defined cell number by using the standard plate as a reference. Each experiment was repeated at least twice, and the mean was plotted. The two-way ANOVA + Bonferroni posttest, comparing each condition to the control-treated cells over time, was used to determine statistically significant differences.

#### BrdU Incorporation and Whole Mount Analysis

To assay for proliferation in whole mounts, SE were incubated with BrdU for 6 h, a quarter of the SE was transferred into PBS and fixed in 3.7% formadehyde/PBS for 2 h at room temperature (RT). After 3 washing steps in PBS, the tissue was stored at 4°C in PBS containing 0.02% sodium azide until further use. To quantify proliferation, samples were treated with 2 M HCl for 25 min followed by washing and further treatment as described (55). The whole mounts were embedded in fluorescent mounting medium and analyzed using a Cell Observer fluorescence microscope equipped with an AxioCam MRm and ZEN software (Zeiss, Jena, Germany). Each whole mount was entirely imaged, and the number of all cells (Hoechst positive) and BrdU-positive cells was determined with Fiji software to calculate the percentage of proliferation. The mean proliferation of 5,000 cells per data point and SE was plotted. Statistical significance was calculated by one-way ANOVA + Dunnett’s multiple comparison test.

### Histological Processing

Specimens were fixed in 3.7% buffered formaldehyde for at least 24 h before dehydrating in graded alcohol and embedding in paraffin. A total of 4 µm sections were mounted to glass slides, dried o/n at 45°C and deparaffinized by two washes in xylene (8 min) followed by stepwise incubation in ethanol [96%, 80%, 70% (4 min each)] and finally in demineralized water. Routinely, they underwent a standard staining procedure with hematoxylin and eosin afterwards.

### Immunofluorescence and Immunohistochemistry

*Immunofluorescence detection of antigens on cryosections*. In total, 6-µm sections of the frozen specimens were fixed in 80% methanol at 4°C for 5 min followed by absolute acetone at −20°C for 2 min. The air-dried sections were incubated in 12% BSA in PBS at RT for 30 min, before primary antibodies diluted in PBS with 3% BSA were applied for an overnight incubation at 4°C. The mono- and polyclonal antibodies utilized are listed in **Supplementary Tables S1** and **S2**. After intermediate washing, appropriate combinations of fluorochrome-conjugated secondary antibodies were added together with 0.2 µg 4′,6-diamidino-2-phenylindole (DAPI) per ml for nuclear staining. After 1 h incubation at RT, the sections were washed and mounted in fluorescent mounting medium (Dako, Glostrup, Denmark). Fluorescent images were recorded at an Olympus AX-70 microscope equipped with epifluorescence illumination, an OSIS F-View CCD camera and the accompanying cell˄R software (Olympus, Shinjuku, Japan).

*Immunohistochemical analyses on histological sections and whole mount specimens of fdmSE*. For staining of whole mount specimens, the tissue was fixed in 2% formaldehyde for 2 h, permeabilized in 0.2% Triton-X in phosphate-buffered saline (PBS) for 15 min, and blocked in blocking buffer (5% donkey serum (Dianova, Hamburg, Germany), 5% goat serum (Dianova), 5% BSA in PBS) for 1 h. Primary antibodies were incubated overnight at 4°C in blocking buffer followed by fluorophore-conjugated secondary donkey antibodies and 2 µg/ml DAPI (Sigma-Aldrich, Taufkirchen, Germany) incubation for 2 h at RT. The whole mounts were mounted in fluorescent mounting medium (Dako). Images were taken with the confocal Leica TCS SP5 II (Leica, Wetzlar, Germany). Samples were analyzed at ×40 magnification and images of 1,024 × 1,024 pixels with a pixel size of 0.4 µm generated. Z-stacks were imaged at intervals of 0.7 µm.

*Immunohistochemistry *histological sections were processed as described for cryosections. For detection of color substrate in transmitting light microscopy, however, the secondary antibodies were conjugated to peroxidase (EnVision HRP Rabbit/Mouse, Dako) and an incubation step with DAB was included. Thereafter, the sections were counterstained with hematoxylin and dehydrated and mounted in Eukitt mounting medium (Sigma-Aldrich, St. Louis, MO, USA). Images were taken using an Olympus-AX-51 microscope equipped with OSIS-Color-View CCD camera and cell˄D software (Olympus).

### Picrosirius Red Staining for Collagen

For the presentation of collagen fibers, histological sections were stained with Picrosirius red according to the protocol of the manufacturer (MORPHISTO, Frankfurt, Germany) and as described in detail previously (20). Sections were examined at a Zeiss-Axiophot-microscope equipped with modules generating circular polarized light. Images were taken using a Zeiss-AxioCam MRc camera and Zeiss AxioVision 4.8.2 software. In order to quantify the amount of fibrillar collagen micrographs of the different specimens (*n* = 3 for the different treatment modalities) were recorded with identical settings during polarization microscopy. The resulting image files were evaluated using NIH ImageJ for integrated signal density in the entire stromal area of the sections. Correction was performed by subtraction of integral background intensity in each individual section. Data are presented as means with standard deviation.

### Gelatinase Assay (*In Situ* Zymography)

MMP-activity *in situ* was visualized in unfixed cryosections using DQ-gelatin (EnzCheck Gelatinase/Collagenase Assay Kit, Live Technologies) as substrate. By proteolytic cleavage, quenched fluorescence is released and becomes detectable. After 1 h incubation at RT with 50 µg/ml DQ-gelatin together with 5 µg/ml Hoechst 33258, the sections were washed and methanol/acetone fixed before mounting in Dako fluorescent mounting medium. The sections were immediately examined using an Olympus AX-70 fluorescence microscope equipped with a OSIS F View CCD camera and the accompanying cell^R software (Olympus). Fluorescence was documented using equal exposure times for all sections.

### Enzyme-Linked Immunosorbent Assay

Matrix metalloproteinase (MMP) secretion was quantified by R&D Quantikine ELISA according to the manufacturer’s protocol. The Pro-MMP-1 ELISA is specific for the Pro-MMP-1 only, whereas the MMP-3 and MMP-9 ELISA detect both the pro- and active forms. All assays were performed with a 48-h conditioned culture medium from 2- or 4-week-old SEs. Duplicate cultures were analyzed with technical replica.

### Western Blot

Cells were lysed with RIPA buffer for 30 min on ice. After centrifugation for 30 min at 14,000 rpm, the protein content of the supernatant was determined using the Pierce BCA Protein assay (Thermo Fischer Scientific). Proteins were separated on a 10% SDS gel and blotted onto a nitrocellulose membrane. After blocking with 5% skim milk in 0.1% PBS-T (blocking buffer) for 1 h, proteins were detected with the respective primary antibody (diluted in PBS-T) overnight at 4°C, the respective HRP-coupled secondary antibody (in blocking buffer) for 1 h at RT and subsequently identified by luminometric detection with ECL (GE, Buckinghamshire, UK).

### Real-Time/Quantitative RT-PCR

For quantitative RT-PCR (qRT-PCR), the Universal Probe Library (UPL) system (Roche, Basel, Switzerland) was used and qRT-PCR was performed in a 96-well plate-based LightCycler 480 Instrument II (Roche) according to the manufacturer’s instructions. The UPL tool (www.universalprobelibrary.com) was used for primer design. For each reaction, a 15-µl mix in nuclease-free water containing 10 µl LightCycler master (2×), 0.4 µM forward and reverse primers (stock: 10 µM), and 0.1 µM UPL probe (stock: 10 µM) were used and 50 ng cDNA in 5 µl RNase-free water was added per well. A negative control (water) was run with each primer pair. Each qRT-PCR was carried out in technical duplicates. The reaction was performed in PCR 96-well TW-MT Plate white (Biozym, Hessisch Oldendorf, Germany) and sealed with Adhesive Clear qPCR Seals (Biozym) with the following protocol: preincubation for 10 min at 95°C, 45 cycles of 10 s at 95°C (ramp rate 4.4°C/s), 30 s at 60°C (ramp rate 2.2°C/s), and 1 s at 72°C (ramp rate 4.4°C/s). Fluorescence was measured after each cycle. Standard curves were performed for each primer pair using a dilution series of 100, 20, 4, 0.80, and 0.16 ng pooled cDNA for each target cell type.

For relative quantification, the gene of interest (target) and the housekeeping gene GAPDH (reference) were compared for each control and stimulated sample. For this calculation, the crossing point value (CP) of each gene in a given sample, identifying the cycle number when fluorescence signals rise above threshold fluorescence, was obtained using the Second Derivative Maximum method of the LightCycler 480 Software (Roche). With these CP values, the ratio of relative gene expression of control vs. treated sample, normalized to GAPDH, was calculated as described (56). Data were displayed using linear or logarithmic scales, whereas the corresponding control was always set to one.

For primers and probes, see **Supplementary Table S3**.

### Multiplex Fluorescence *In Situ* Hybridization

M-FISH was performed as described by Geigl etal. (57). Briefly, seven pools of flow-sorted human chromosome painting probes were amplified and directly labeled using seven different fluorochromes (DEAC, FITC, Cy3, Cy3.5, Cy5, Cy5.5, and Cy7) by degenerative oligonucleotide-primed PCR (DOP-PCR). Metaphase spreads immobilized on glass slides were denatured in 70% formamide/2xSSC pH 7.0 at 72°C for 2 min followed by dehydration in a degraded ethanol series. Hybridization mixture containing combinatorically labeled painting probes, an excess of unlabeled cot1 DNA, 50% formamide, 2xSSC, and 15% dextran sulfate were denatured for 7 min at 75°C, preannealed at 37°C for 20 min, and hybridized at 37°C to the denaturated metaphase preparations. After 48 h, the slides were washed in 2xSSC at room temperature for 3 × 5 min followed by two washes in 0.2xSSC/0.2% Tween-20 at 56°C for 7 min, each. Metaphase spreads were counterstained with DAPI and covered with antifade solution. Metaphase spreads were recorded using a DM RXA epifluorescence microscope (Leica Microsystems, Bensheim, Germany) equipped with a Sensys CCD camera (Photometrics, Tucson, AZ, USA). Camera and microscope were controlled by the Leica Q-FISH software, and images were processed based on the Leica MCK software and presented as multicolor karyograms (Leica Microsystems Imaging solutions, Cambridge, UK).

### Statistical Analysis

Statistical significance was calculated by performing a one-way ANOVA + Dunnett’s multiple comparison test or two-way ANOVA + Bonferroni posttest, which both compare each treatment modality to the corresponding controls. The *p*-values as well as the used test are indicated within the figure or legend, respectively. The analyses were performed by GraphPad Prism 4 software.

## Data Availability Statement

The original contributions presented in the study are included in the article/**Supplementary Material**, further inquiries can be directed to the corresponding author.

## Ethics Statement

The studies involving human participants were reviewed and approved by the East London and City Health Authority local ethics committee. The protocol was approved by the East London and City Health Authority local ethics committee. The patients/participants provided their written informed consent to participate in this study.

## Author Contributions

MT and H-JS contributed equally in all aspects of the work. AJ contributed to the genetic analyses. CH contributed to the patients’ tumor material. EPL contributed to the writing and submission. PB has contributed to all aspects of the work and led the team effort. All authors contributed to the article and approved the submitted version.

## Funding

This work was supported by contract research “Adulte epidermale Stammzellen” of the Baden-Württemberg Stiftung (P-BWS-ASII/35) and a grant from the Federal Ministry for Research and Education/BMBF (KAUVIR, 02NUK036A) (all to PB).

## Conflict of Interest

The authors declare that the research was conducted in the absence of any commercial or financial relationships that could be construed as a potential conflict of interest.

## Publisher’s Note

All claims expressed in this article are solely those of the authors and do not necessarily represent those of their affiliated organizations, or those of the publisher, the editors and the reviewers. Any product that may be evaluated in this article, or claim that may be made by its manufacturer, is not guaranteed or endorsed by the publisher.

## Supplementary Material

Supplementary Figure S1 Vemurafenib does not cause major chromosomal instability. HaCaT cells were treated with either DMSO or Vemurafenib (1μM or 5μM) for 5 weeks and analyzed by M-FISH for their chromosomal status. **(A)** examples of M-FISH karyograms of control HaCaT cells and HaCaT cells treated with 5 µM Vemurafenib for 5 weeks. **(B)** The comparison of subpopulation distribution showed a shift in superiority of pre-existing populations. Graph indicates pre-existing subpopulations and their shifts in low (1μM, yellow) or high (5μM, green) concentration of Vemurafenib.

Supplementary Figure S2 Characterization of Vemurafenib’s effects on epithelial differentiation in HaCaT SEs. SEs from HaCaT cells were treated with Vemurafenib (1 µM and 5 µM) for up to 5 weeks and histology and immunostaining was performed at the indicated time points. **(A)** H&E staining of SEs demonstrate improved tissue organization with an epidermis-like stratification and improved parakeratotic str. corneum, particularly evident upon 5µM Vemurafenib. In addition, Vemurafenib-treated HaCaT SEs show extended vitality, as evidenced by an increased number of vital basal cells and improved attachment of the epithelium to the DE. **(B)** Improved and more normal differentiation in HaCaT SEs treated with Vemurafenib for 3 weeks. Immunostaining for the early KRT10 and particularly the late differentiation markers FLG and KRT2, point to improved differentiation including a more structured str. granulosum. The BM components COLVII (green), COLIV (red), and LAM (red) are concentrated in the continuous BMs. Note that COLIV is continuously expressed by the fibroblasts and present throughout the DE, though enriched in the BM zone (left panel). LAM, that appears “bloated” in the control SEs, becomes more concentrated upon Vemurafenib treatment. **(C)** Immunohistochemical analysis of the SEs demonstrate a strong increase of pERK1/2 in the vital cell layers of the Vemurafenib-treated (5 µM for 3 weeks, right) SEs, as compared to the control SEs (left). Scale bar = 300 µm in **(A, C)** and 150 µm in **(B)**.

Supplementary Figure S3 pERK1/2 expression in Vemurafenib (5 µM)-treated SEs. In NHEK SEs pERK1/2 is expressed in the epidermis and prominently also in the dermal fibroblasts. COLVII is expressed all along the BM (upper panel). In HrasA5 SEs, expression of pERK1/2 is increased in the entire epithelium and particularly dominant in the invasive strands. COLVII is lost at the invasive sites (middle panel). Unphosphorylated ERK1/2 (control) is expressed throughout the epithelium in control and Vemurafenib-treated SEs. The dermal fibroblasts are depicted by counterstaining for Vimentin. (lower panel). Scale bar = 300 μm.

Supplementary Figure S4 Action profile of Cobimetinib in HrasA5-SEs. **(A)** H&E staining of HrasA5-SEs treated for 3 weeks with the MEK-inhibitor Cobimetinib illustrates a toxic effect with dose-dependent tissue atrophy. 0.1 µM Cobimetinib causes an only mild structural impairment (middle) while 1.0 µM leads to strong atrophy (lower). Scale bar = 300 µm. **(B)** Western Blot analysis of a competition experiment with cultured HrasA5-cells treated with Vemurafenib in combination with Cobimetinib reveals a dose-dependent pMEK 1/2 accumulation and complete pERK 1/2 inhibition. **(C)** Vemurafenib- and Vemurafenib + Cobimetinib-treated HrasA5-monolayer cultures were subjected to mRNA expression analysis of a panel of genes that had been identified as Vemurafenib-responsive. All genes, including the main players in the degradome, MMP1 and MMP3, were strongly repressed in the presence of Cobimetinib. Normalization was performed using GAPDH and fold-changes were expressed by comparing each treatment to the DMSO control set to one.; n=2, mean ± SD, log 2-scale.
