# Supplementary figures and images for: Adverse Effects of Vemurafenib on Skin Integrity: Hyperkeratosis and Skin Cancer Initiation Due to Altered MEK/ERK-Signaling and MMP Activity
Source: Front Oncol. 2022 Jan 31;12:827985. doi: 10.3389/fonc.2022.827985 (PMC8842679; doi:10.3389/fonc.2022.827985)

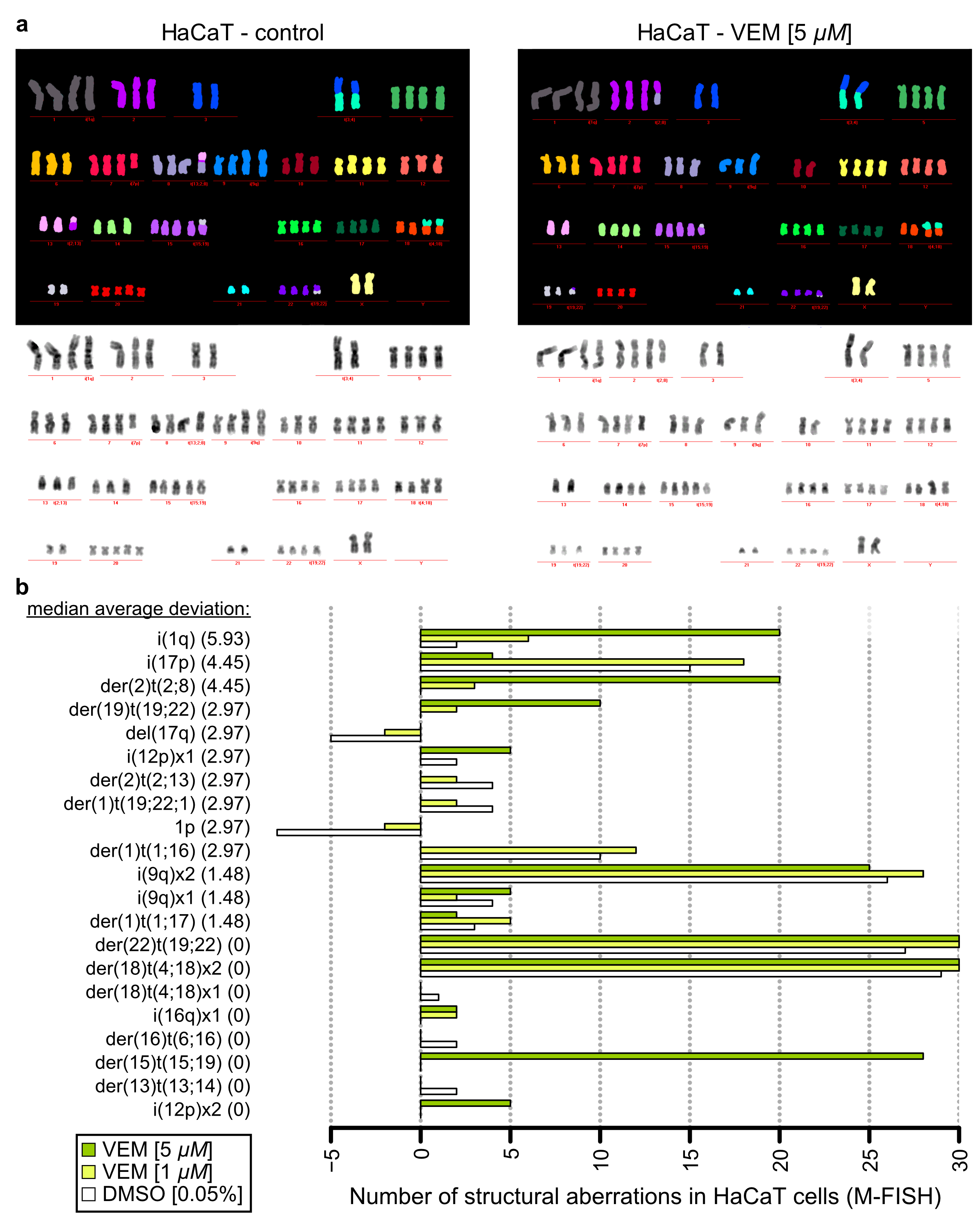

Supplement: Supplementary Figure S1 — Vemurafenib does not cause major chromosomal instability. HaCaT cells were treated with either DMSO or Vemurafenib (1μM or 5μM) for 5 weeks and analyzed by M-FISH for their chromosomal status. (A) examples of M-FISH karyograms of control HaCaT cells and HaCaT cells treated with 5 µM Vemurafenib for 5 weeks. (B) The comparison of subpopulation distribution showed a shift in superiority of pre-existing populations. Graph indicates pre-existing subpopulations and their shifts in low (1μM, yellow) or high (5μM, green) concentration of Vemurafenib. [file Image_1.tiff]

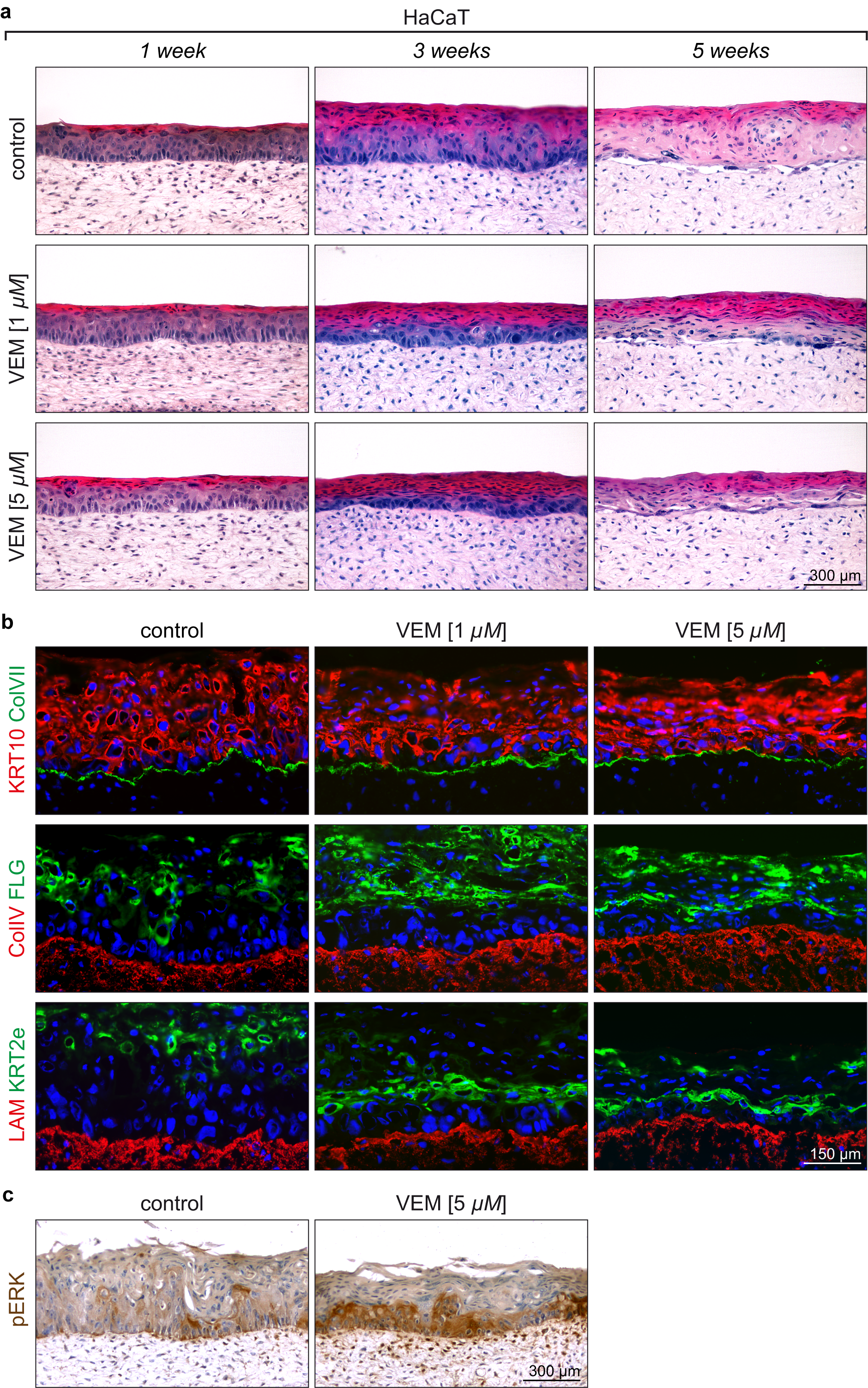

Supplement: Supplementary Figure S2 — Characterization of Vemurafenib’s effects on epithelial differentiation in HaCaT SEs. SEs from HaCaT cells were treated with Vemurafenib (1 µM and 5 µM) for up to 5 weeks and histology and immunostaining was performed at the indicated time points. (A) H&E staining of SEs demonstrate improved tissue organization with an epidermis-like stratification and improved parakeratotic str. corneum, particularly evident upon 5µM Vemurafenib. In addition, Vemurafenib-treated HaCaT SEs show extended vitality, as evidenced by an increased number of vital basal cells and improved attachment of the epithelium to the DE. (B) Improved and more normal differentiation in HaCaT SEs treated with Vemurafenib for 3 weeks. Immunostaining for the early KRT10 and particularly the late differentiation markers FLG and KRT2, point to improved differentiation including a more structured str. granulosum. The BM components COLVII (green), COLIV (red), and LAM (red) are concentrated in the continuous BMs. Note that COLIV is continuously expressed by the fibroblasts and present throughout the DE, though enriched in the BM zone (left panel). LAM, that appears “bloated” in the control SEs, becomes more concentrated upon Vemurafenib treatment. (C) Immunohistochemical analysis of the SEs demonstrate a strong increase of pERK1/2 in the vital cell layers of the Vemurafenib-treated (5 µM for 3 weeks, right) SEs, as compared to the control SEs (left). Scale bar = 300 µm in (A, C) and 150 µm in (B). [file Image_2.tif]

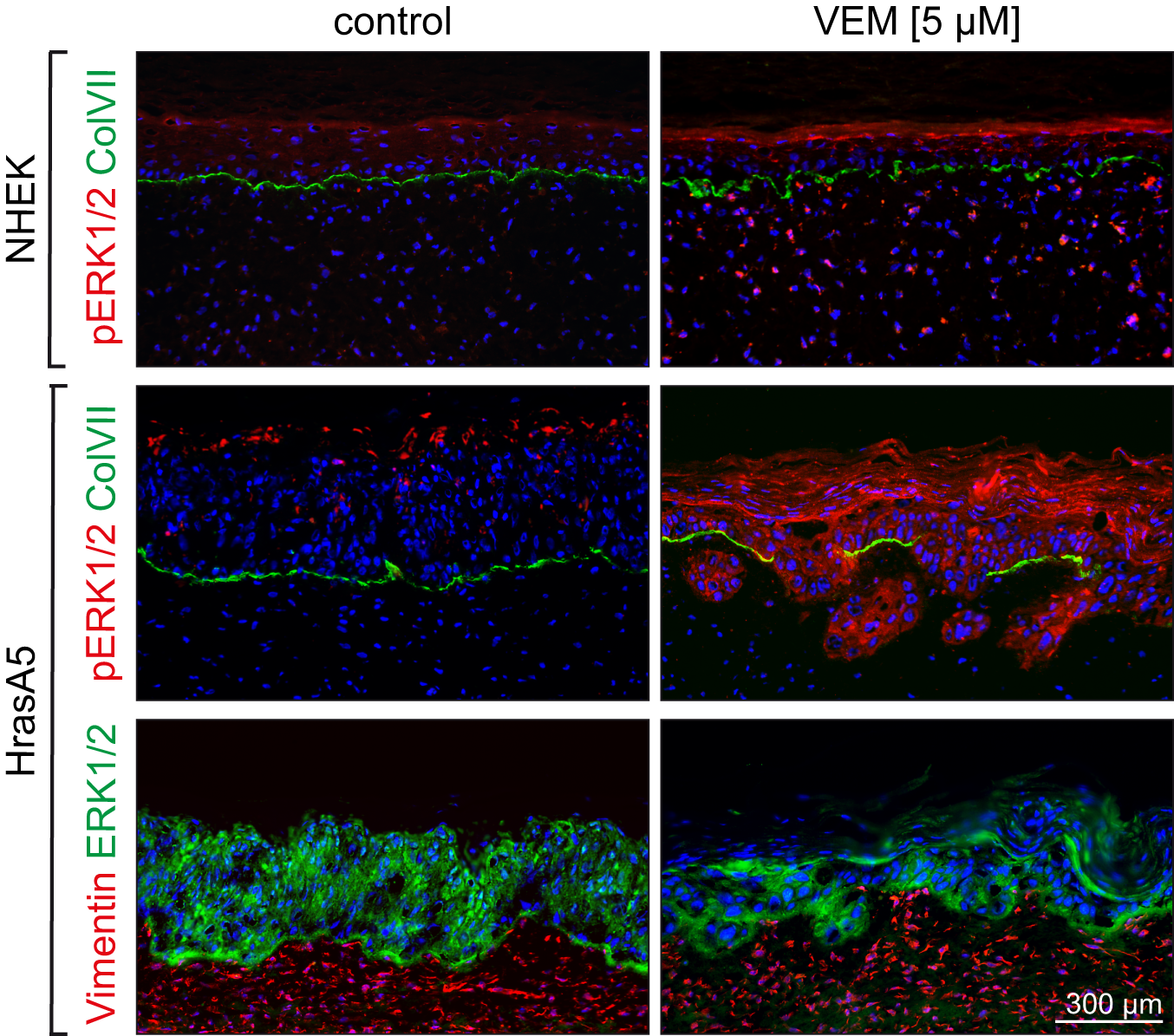

Supplement: Supplementary Figure S3 — pERK1/2 expression in Vemurafenib (5 µM)-treated SEs. In NHEK SEs pERK1/2 is expressed in the epidermis and prominently also in the dermal fibroblasts. COLVII is expressed all along the BM (upper panel). In HrasA5 SEs, expression of pERK1/2 is increased in the entire epithelium and particularly dominant in the invasive strands. COLVII is lost at the invasive sites (middle panel). Unphosphorylated ERK1/2 (control) is expressed throughout the epithelium in control and Vemurafenib-treated SEs. The dermal fibroblasts are depicted by counterstaining for Vimentin. (lower panel). Scale bar = 300 μm. [file Image_3.tif]

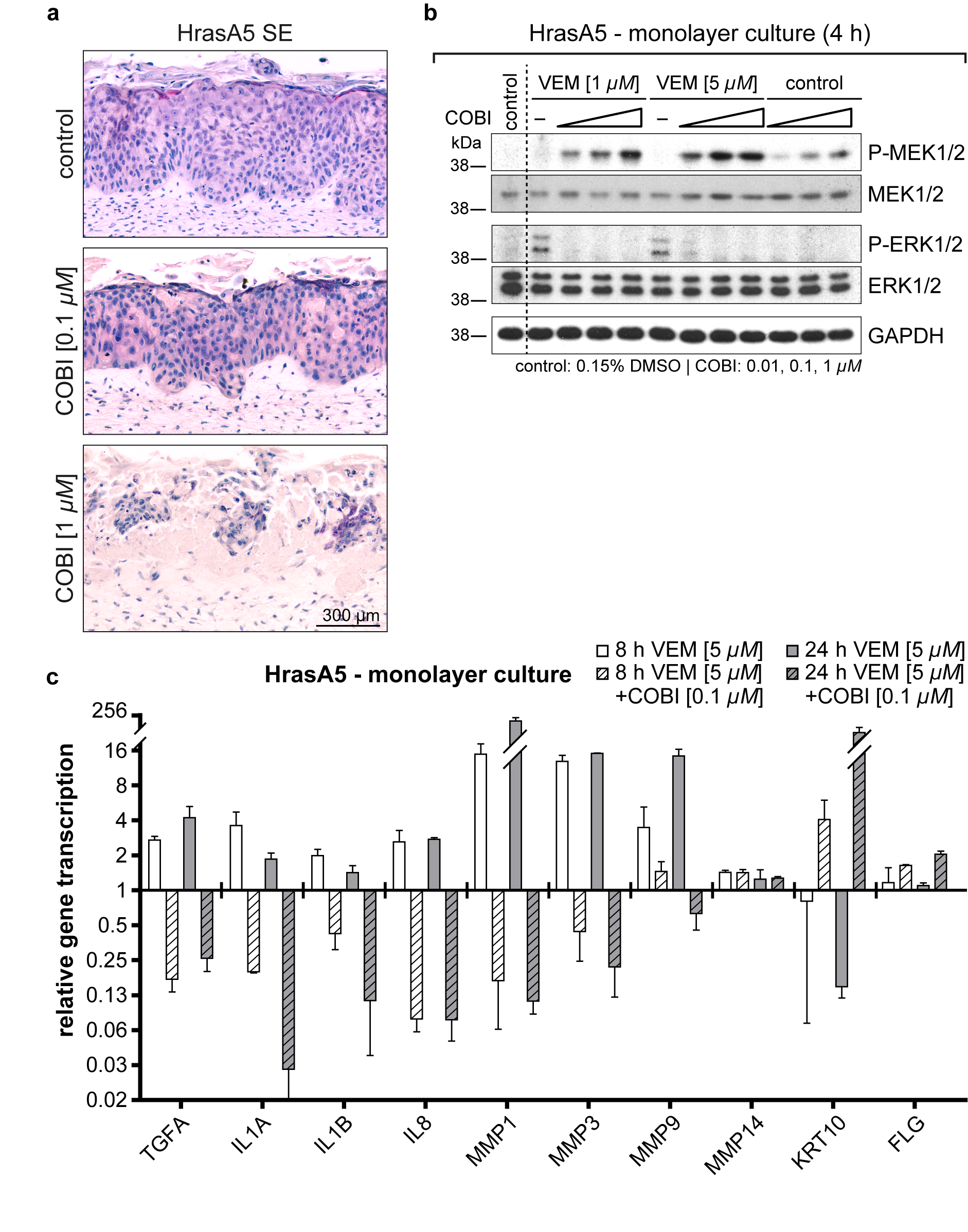

Supplement: Supplementary Figure S4 — Action profile of Cobimetinib in HrasA5-SEs. (A) H&E staining of HrasA5-SEs treated for 3 weeks with the MEK-inhibitor Cobimetinib illustrates a toxic effect with dose-dependent tissue atrophy. 0.1 µM Cobimetinib causes an only mild structural impairment (middle) while 1.0 µM leads to strong atrophy (lower). Scale bar = 300 µm. (B) Western Blot analysis of a competition experiment with cultured HrasA5-cells treated with Vemurafenib in combination with Cobimetinib reveals a dose-dependent pMEK 1/2 accumulation and complete pERK 1/2 inhibition. (C) Vemurafenib- and Vemurafenib + Cobimetinib-treated HrasA5-monolayer cultures were subjected to mRNA expression analysis of a panel of genes that had been identified as Vemurafenib-responsive. All genes, including the main players in the degradome, MMP1 and MMP3, were strongly repressed in the presence of Cobimetinib. Normalization was performed using GAPDH and fold-changes were expressed by comparing each treatment to the DMSO control set to one.; n=2, mean ± SD, log 2-scale. [file Image_4.tif]
