# Supplementary material for: Adverse Effects of Vemurafenib on Skin Integrity: Hyperkeratosis and Skin Cancer Initiation Due to Altered MEK/ERK-Signaling and MMP Activity
Source: Front Oncol. 2022 Jan 31;12:827985. doi: 10.3389/fonc.2022.827985 (PMC8842679; doi:10.3389/fonc.2022.827985)
Supplement: Supplementary file 5 [file Table_1.docx]

**Table S1 Primary antibodies.**

Specificity Species Dilution Company Cat. -No.

Akt, phosphor (Thr308) Rabbit 1:1,000 Cell Signaling 9275

alpha-SMA Mouse 1:100 Sigma-Aldrich A2547

BrdU Mouse 1:5 BD Biosciences 347580

CD3-ζ Mouse 1:100 Santa Cruz sc-1239

Collagen IV Rabbit 1:100 Progen 10760

Collagen VII Mouse 1:400 Sigma-Aldrich C6805

ERK1/2 Rabbit 1:2,000 Cell Signaling 9102

ERK1/2, phosphor (Thr202/ Tyr204) Mouse 1:2,000 Cell Signaling 9106

ERK1/2, phosphor (Thr202/ Tyr204) Rabbit 1:400 Cell Signaling 4370

Filaggrin Mouse 1:200 Acris Antibodies AM00245

GAPDH Mouse 1:50,000 Merck Millipore MAB374

GM-CSF Mouse 1:100 Novus NB100-65022

HIS10ph Rabbit 1:500 Abcam Ab32107

Ki67 Rabbit 1:300 Abcam ab15580

KRT2 Mouse 1:250 Progen 65191

KRT10 Mouse 1:20 Progen 11414

KRT15 Guinea pig 1:250 Progen GP-CK15

Laminin 5 (γ2 chain) Mouse 1:200 Merck Millipore MAB19562

MEK1/2 Mouse 1:1,000 Cell Signaling 4694

MEK1/2, phosphor (Ser217/ Ser221) Rabbit 1:1,000 Cell Signaling 9154

MMP-1 Rabbit 1:100 Sigma-Aldrich M3940

MMP-3 Rabbit 1:200 Bender Med Systems 4423

p38 alpha Mouse 1:2,000 Cell Signaling 9217

p38, phosphor (Thr180/ Tyr182) Rabbit 1:1,000 Cell Signaling 9211

Vimentin Mouse 1:100 Progen 61013

TGM Mouse 1:20 CellSystems 5003

**Table S2 Secondary antibodies.**

Specificity Species Conjugate Dilution Company Cat. -No.

Digoxigenin (DIG) Sheep Horseradish peroxidase 1:300 Roche Applied Sciences 11207733910

Mouse-IgG Goat Alexa-488 1:600 Life Technologies A11029

Mouse-IgG Goat Cy3 1:500 Jackson ImmunoResearch 115-165-205

Rabbit-IgG Goat Cy3 1:600 Dianova 111-165-144

Rat-IgG Donkey Cy3 1:500 Dianova 712-166-153

Mouse-IgG Donkey Horseradish peroxidase 1:40,000 Dianova 715-035-150

**Table S3 qRT-PCR primers**

Gene Primer sequences (5’-3’) UPL # Efficiency Cell type

*alphaSMA*  ccctgaagtacccgatagaaca (fwd)

ggcaacacgaagctcattg (rev) 9 1.94 Fibroblasts

*AXIN2* cagccattcaggaactaccc (fwd) 3 1.93 Fibroblasts

gaaggtgtgtggaggaaagg (rev) 1.99 Keratinocytes

*CCL2*  agtctctgccgcccttct (fwd)

gtgactggggcattgattg (rev) 40 1.97 Fibroblasts

*CDKN1A*  tcactgtcttgtacccttgtgc (fwd) 32 1.94 Fibroblasts

ggcgtttggagtggtagaaa (rev) 1.99 Keratinocytes

*CSF2* tctcagaaatgtttgacctcca (fwd)

gcccttgagcttggtgag (rev) 1 2.00 Fibroblasts

*CXCL10*  gaaagcagttagcaaggaaaggt (fwd)

gacatatactccatgtagggaagtga (rev) 34 2.00 Fibroblasts

*CXCL12* gctggtcctcgtgctgac (fwd)

gcatgggcatctgtagctc (rev) 13 1.86 Fibroblasts

*EGFR* ctatgtgcagaggaattatgatcttt (fwd)

gggcaatgaggacataacca (rev) 79 2.00 Keratinocytes

*FGF7* aagggacccaagagatgaaga (fwd)

cctttgattgccacaattcc (rev) 59 2.00 Fibroblasts

*FGF10* gaaggagaactgcccgtaca (fwd)

ggcaacaactccgatttctact (rev) 80 2.00 Fibroblasts

*FLG*  ggactctgagaggcgatctg (fwd)

tgctcccgagaagatccat (rev) 38 1.98 Keratinocytes

*GAPDH*  agccacatcgctcagacac (fwd) 60 1.93 Fibroblasts

gcccaatacgaccaaatcc (rev) 1.97 Keratinocytes

*HGF*  cagcatgtcctcctgcatc (fwd)

tcttttcctttgtccctctgc (rev) 15 1.99 Fibroblasts

*IL1A* ggttgagtttaagccaatcca (fwd) 6 2.00 Fibroblasts

tgctgacctaggcttgatga (rev) 1.98 Keratinocytes

*IL1B*  tacctgtcctgcgtgttgaa (fwd) 78 1.94 Fibroblasts

tctttgggtaatttttgggatct (rev) 1.97 Keratinocytes

*IL6*  gatgagtacaaaagtcctgatcca (fwd)

ctgcagccactggttctgt (rev) 40 1.70 Fibroblasts

*IL8*  agacagcagagcacacaagc (fwd)

atggttccttccggtggt (rev) 72 1.97 Fibroblasts

*IVL*  acccatcaggagcaaatgaa (fwd)

agctcgacaggcaccttct (rev) 16 2.00 Keratinocytes

*KRT10*  ccatcgatgaccttaaaaatcag (fwd)

cgcagagctacctcattctcata (rev) 64 2.00 Keratinocytes

*MMP1* gctaacctttgatgctataactacga (fwd) 7 1.94 Fibroblasts

tttgtgcgcatgtagaatctg (rev) 1.98 Keratinocytes

*MMP3* cagtttgctcagcctatcca (fwd) 58 2.00 Fibroblasts

tcacatctttttcgaggtcgt (rev) 2.00 Keratinocytes

*MMP9* gaaccaatctcaccgacagg (fwd) 6 1.89 Fibroblasts

gccacccgagtgtaaccata (rev) 1.70 Keratinocytes

*MMP14* caggaatgaggatctgaatgg (fwd) 45 2.00 Fibroblasts

ccgaggggtcactggaat (rev) 2.00 Keratinocytes

*SNAI2* tggttgcttcaaggacacat (fwd)

gcaaatgctctgttgcagtg (rev) 7 1.95 Keratinocytes

*TGFA*  cccagattcccacactcag (fwd) 38 2.00 Fibroblasts

acgtacccagaatggcagac (rev) 1.99 Keratinocytes

*TGFB1*  gcagcacgtggagctgta (fwd) 72 1.98 Fibroblasts

cagccggttgctgaggta (rev) 2.00 Keratinocytes

*TGFB3* aagaagcgggctttggac (fwd) 38 2.00 Fibroblasts

gcgcacacagcagttctc (rev) 1.93 Keratinocytes

All primers were derived from Sigma-Aldrich (Taufkirchen, Germany).
